# scATD: a high-throughput and interpretable framework for single-cell cancer drug resistance prediction and biomarker identification

**DOI:** 10.1093/bib/bbaf268

**Published:** 2025-06-12

**Authors:** Murong Zhou, Zeyu Luo, Yu-Hang Yin, Qiaoming Liu, Guohua Wang, Yuming Zhao

**Affiliations:** College of Computer and Control Engineering, Northeast Forestry University, Harbin 150040, China; College of Life Science, Northeast Forestry University, Harbin 150040, China; College of Computer and Control Engineering, Northeast Forestry University, Harbin 150040, China; College of Computer and Control Engineering, Northeast Forestry University, Harbin 150040, China; College of Life Science, Northeast Forestry University, Harbin 150040, China; College of Artificial Intelligence, Henan University, Zhengzhou 450000, China; College of Computer and Control Engineering, Northeast Forestry University, Harbin 150040, China; College of Computer and Control Engineering, Northeast Forestry University, Harbin 150040, China

**Keywords:** large language model (LLM), cancer drug resistance, single-cell RNA sequencing, knowledge distillation, model interpretation

## Abstract

Transfer learning has been widely applied to drug sensitivity prediction based on single-cell RNA sequencing, leveraging knowledge from large datasets of cancer cell lines or other sources to improve the prediction of drug responses. However, previous studies require model fine-tuning for different patient single-cell datasets, limiting their ability to meet the clinical need for high-throughput rapid prediction. In this research, we introduce single-cell Adaptive Transfer and Distillation model (scATD), a transfer learning framework leveraging large language models for high-throughput drug sensitivity prediction. Based on different large language models (scFoundation and Geneformer) and transfer strategies, scATD includes three distinct sub-models: scATD-sf, scATD-gf, and scATD-sf-dist. scATD-sf and scATD-gf employs an important bidirectional style transfer to enable predictions for new patients without model parameter training. Additionally, scATD-sf-dist uses knowledge distillation from large models to enhance prediction performance, improve efficiency, and reduce resource requirements. Benchmarking across more diverse datasets demonstrates scATD’s superior accuracy, generalization and efficiency. Besides, by rigorously selecting reference background samples for feature attribution algorithms, scATD also provides more meaningful insights into the relationship between gene expression and drug resistance mechanisms. Making scATD more interpretability for addressing critical challenges in precision oncology.

## Introduction

Understanding drug responses at the single-cell level in cancer is crucial in clinical oncology, due to the inherent heterogeneity of tumor cells that drives drug resistance and treatment failure. Tumor cells that survive initial drug treatments can evolve into resistant subpopulations, complicating therapeutic interventions. Identifying drug responses at the single-cell level offers insights into tumor heterogeneity, drug resistance mechanisms, and drug-induced tumor evolution [1]. Advances in RNA sequencing technologies [2] have facilitated the accumulation of bulk gene expression datasets with drug response labels. These datasets have enabled the development of predictive models for drug responses [3]. Single-cell RNA sequencing (scRNA-seq), which provides cell-specific insights, has further spurred interest in modeling drug responses at the single-cell level. However, the scarcity of high-quality single-cell drug response labels has posed a significant challenge [4].

Domain adaptation frameworks such as SCAD [4], scDEAL [5], and scAdaDrug [6] address this limitation by transferring drug response knowledge from bulk tissue data to single-cell data. These methods achieve high-precision predictions without requiring labeled single-cell datasets for training. Meanwhile, pretraining Large Language models (LLMs) on extensive unlabeled datasets and fine-tuning them with limited domain-specific data has emerged as a dominant paradigm for overcoming label scarcity [7, 8]. LLMs leverage extensive pretraining to learn robust prior representations, enabling downstream generalization to unseen datasets. In transcriptomics, Pretrained LLMs have shown efficacy in modeling RNA-seq data and are utilized in various transcriptomics tasks [9–12]. Domain-specific models, such as scFoundation [10] and Geneformer [11], exemplify the power of LLMs in RNA-seq tasks. For instance, scFoundation, integrated with SCAD, improves single-cell drug response prediction accuracy. Notably, although scFoundation was not pre-trained on bulk RNA-seq datasets, its representational space closely aligns with both bulk and single-cell data, a feature traditionally sought in small adaptive transfer learning models. This adaptability highlights the potential of such LLM-based architectures to bridge representational gaps across diverse data modalities, thereby advancing predictive capabilities in RNA-seq related tasks.

However, existing models, regardless of model size, depend on supervised training or domain-adaptive transfer learning to retrain the network parameters after pretraining to fit the drug response prediction task for new patients. Even bulk models are constructed on one type of drug-cancer; the inherent variability in single-cell gene expression patterns across patients highlights the necessity of retraining models to improve prediction accuracy for new patients. Combining the increasing parameter size of large models exacerbates issues of inference latency [8]. These factors pose challenges for meeting clinical demands for high-throughput detection of different patients’ drug responses at the single-cell level. Additionally, with the rapid accumulation of single-cell drug response datasets containing high-quality labels, it is time to re-evaluate the necessity of unsupervised transfer learning methods, as recent studies directly supervised training at the single-cell RNA-seq data [13].

Interpretability remains another critical challenge in deploying these models to foster trust in model predictions within clinical applications [14, 15]. While feature attribution methods have been employed to identify genes that are important for drug response predictions, recent studies highlight the risks of over-reliance on single interpretability techniques without robust validation [16, 17]. Moreover, most previous investigations have focused solely on global interpretability to evaluate gene importance rankings and identify key genes [4, 5] while largely overlooking local interpretability analyses that assess gene contribution patterns at the individual patient level. This gap limits our understanding of patient-specific drug resistance mechanisms. More urgently, related work lacks guidelines for selecting reference background samples. Our experiments reveal significant gene interpretation issues when using default zero baselines.

**Figure 1:**
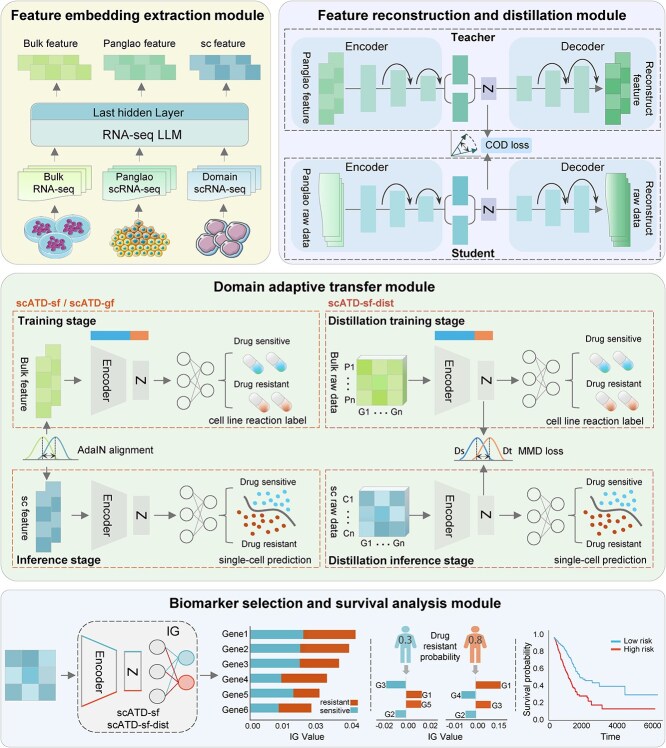
Overall framework of scATD. scATD include four main modules: (a) feature embedding extraction module. (b) feature reconstruction and distillation module. (c) domain adaptive transfer module. (d) biomarker selection and survival analysis module. For Adain alignment transfer learning, bulk data transfer to Panglao data during model training, and patient single cell data transfer to bulk during model inference. Alt text: Schematic overview of the scATD framework consisting of four main modules. (a) Feature embedding extraction module: Bulk, Panglao, and single-cell RNA-seq data are processed through an RNAseq large language model (LLM), extracting embeddings from the last hidden layer. (b) feature reconstruction and distillation module: A teacher-student autoencoder framework reconstructs Panglao features and raw data, with COD loss guiding knowledge distillation from teacher to student. (c) domain adaptive transfer module: For scATD-sf/gf, the bulk features and cell line drug response labels are used in training via Bi-AdaIN alignment; single-cell features are used for inference. For scATD-sf-dist, a two-stage distillation uses bulk raw data in training and single-cell raw data in inference, with MMD loss aligning feature distributions. (d) biomarker selection and survival analysis module: Integrated gradients (IG) identify important genes for drug resistance prediction. Bar plots show IG values, dot plots show patientspecific predictive gene contributions, and a Kaplan–Meier curve shows survival difference between highand low-risk groups.

To tackle these challenges, we propose the scATD framework (Fig. 1). scATD integrates bulk and single-cell data into a unified Residual Variational Autoencoder (Res-VAE) architecture, employing a Bi-AdaIN (Bidirectional Adaptive Instance Normalization, we modified the original version [18] to this bidirectional approach) for style transfer and leveraging knowledge distillation to enhance model prediction efficiency and scalability. Specifically, scATD leverages pre-trained LLMs to extract rich feature representations from RNA-seq data, which are subsequently refined during Res-VAE pretraining. Based on features extracted from different pre-trained LLMs, two independent models, scATD-sf and scATD-gf, were trained (details in Methods). This approach enhances latent feature representation and facilitates robust adaptation to single-cell data. The Bi-AdaIN mechanism enables parameter-free style transfer between bulk and single-cell datasets, allowing scATD to predict single-cell drug responses within the same drug-cancer type without necessitating additional retraining, thereby streamlining clinical applicability. Experimental validation of unsupervised representation and predictive performance across diverse independent datasets demonstrates that scATD achieves state-of-the-art (SOTA) predictive accuracy and generalizability across datasets ranging from hundreds to tens of thousands of cells. Moreover, we compare the performance of RNA-seq representations between two foundational LLMs, scFoundation and Geneformer, elucidating their differences in fine-tuning for drug resistance prediction performance.

Given the increasing parameter sizes of LLMs and the resulting inference latency, we implement knowledge distillation techniques [19] to develop the scATD-sf-dist (details in Methods), which embeds the LLMs feature extraction rules into Res-VAE (the backbone of scATD-sf-dist) during pretraining. This approach mitigates computational overhead while preserving predictive accuracy. To enhance interpretability, scATD integrates advanced feature attribution techniques [14, 20] to identify biomarkers at multiple levelsacohort, individual patient, and single cell. These biomarkers including key features and genes are rigorously validated through literature support, cross-dataset robustness testing, and perturbation of feature attribution algorithms, providing insights into drug resistance mechanisms and potential clinical applications. Notably, by leveraging local interpretability analyses, the model reveals the contribution and relationship (approximately linear or nonlinear) of individual gene expression levels to drug resistance.

In summary, our experiment provides new insights into the effective use of RNA-seq LLMs for single-cell drug response prediction, striving not only for high accuracy but also for improved computational efficiency, streamlining model deployment and interpretability, and addressing key challenges in precision medicine.

## Methods

### Datasets

The Panglao dataset, accessible via *PanglaoDB* (https://panglaodb.se/) [21], consists of over one million single-cell expression profiles and was used for pretraining. Additionally, bulk data (cell line data), including 1280 cancer cell lines and 83 drugs/compounds across multiple cancer types, are integrated by retaining shared genes across two databases, Genomics of Drug Sensitivity in Cancer (GDSC) [22] and Cancer Cell Line Encyclopedia (CCLE) [23]. Bulk data are used to provide labels required for supervised learning and serve as source-domain data to establish the mapping relationship from RNA-seq data to drug response labels needed for model transfer. Fourteen single-cell data is sourced from the Gene Expression Omnibus (GEO) database [24], detailed in the Code and Data Availability section. Descriptions of these datasets are provided in Table 1. Single-cell sequencing data from six prostate cancer patients in the GSE137829 datasets were utilized for the model interpretability perturbation experiments of scATD. Furthermore, RNA-seq and corresponding clinical prognosis data were retrieved from The Cancer Genome Atlas (TCGA) database [25, 26], covering five cancer types: lung adenocarcinoma (LUAD), pancreatic adenocarcinoma (PAAD), breast invasive carcinoma (BRCA), skin cutaneous melanoma (SKCM), and prostate adenocarcinoma (PRAD). These datasets were utilized for scATD to identify biomarkers and analyze prognosis risk. Besides, two datasets, namely BRCA_RECIST and PAAD_RECIST, which include RECIST labels, are used to evaluate the model’s generalization performance on the clinically guided drug response prediction task (as detailed in the Code and Data Availability section).

**Table 1:** Information of GEO single cell datasets.

**Drug**	**Dataset**	**Source**	**Cancer type**	**Cell Count**	
				**sensitive**	**resistant**
Cisplatin	GSE117872_HN120	Tumor tissue	Oral squamous cell carcinoma	361	187
Cisplatin	GSE117872_HN137	Tumor tissue	Oral squamous cell carcinoma	403	165
Crizotinib	GSE223779	Tumor organoid	Non-small cell lung cancer	11 153	11 981
Docetaxel	GSE140440	DU145&PC3 cell line	Prostate cancer	162	162
Erlotinib	GSE149214	PC9 cell line	Non-small cell lung cancer	985	1232
Erlotinib	GSE149383	PC9 cell line	Non-small cell lung cancer	1070	1442
Gefitinib	GSE112274	PC9 cell line	Non-small cell lung cancer	37	470
Gefitinib	GSE202234	H1975 cell line	Non-small cell lung cancer	444	400
Gemcitabine	GSE186960	Panc1 cell line	pancreatic cancer	698	5438
Ibrutinib	GSE111014	PBMCs	Chronic lymphocytic leukemia	11 964	12 643
Paclitaxel	GSE163836	FCIBC02 cell line	Breast cancer	1978	5380
Paclitaxel	GSE169246	Tumor tissue	Breast cancer	30 227	36 913
Palbociclib	GSE131984	SUM159 cell line	Breast cancer	1061	693
PLX.4720	GSE108383_A375	A375 cell line	Melanoma	78	52
PLX.4720	GSE108383_X451	451Lu cell line	Melanoma	84	113
PLX.4720	GSE108397	451Lu cell line	Melanoma	3286	3262

Chemotherapy: Cisplatin, Docetaxel, Gemcitabine, Paclitaxel. Targeted therapy: Crizotinib (ALK/ROS1/MET), Erlotinib (EGFR), Gefitinib (EGFR), Ibrutinib (BTK), Palbociclib (CDK4/6), PLX4720 (BRAF V600E).

To ensure reproducibility, all experiments conducted with scATD were performed using a fixed random seed value of 42.

### Overall framework of scATD and comparative models

scATD (single-cell Adaptive Transfer and Distillation model) encompasses four main modules: feature embedding extraction module, feature reconstruction and distillation module, domain adaptive transfer module, and biomarker selection and survival analysis module. The comprehensive structure of the scATD is displayed in Fig. 1.

The proposed framework extracts high-dimensional embeddings of bulk and single-cell data from a pre-trained LLM, followed by a Variational Autoencoder (VAE) [27] that reconstructs features and learns their lower-dimensional latent representations. The VAE model is first pretrained on the PanglaoDB dataset and then incorporated into a domain-adaptive transfer module to align features between bulk and single-cell data. Additionally, a trained distilled VAE (scATD-sf-dist) will also be used independently for downstream drug transfer learning. Finally, interpretability methods assess feature importance and key features (or genes) selection as potential biomarkers.

Notably, several models were developed based on the scATD framework. **scATD-sf** utilizes embeddings from scFoundation as input and integrates Res-VAE with the Bi-AdaIN architecture, while **scATD-gf** employs embeddings from Geneformer with the same architectural components. Additionally, **scATD-sf-dist** takes raw RNA-seq expression profiles as input, adopts Res-VAE as its base architecture, and is trained using knowledge distillation from scATD-sf, with MMD serving as the transfer mechanism. For comparison, we include **scDEAL**, which uses raw RNA-seq expression profiles as input and employs MMD as the transfer architecture; **scFoundation-SCAD**, which is based on the SCAD architecture but utilizes scFoundation embeddings as input and adopts ADDA (Adversarial Discriminative Domain Adaptation) as the transfer mechanism; and **DrugFormer**, a pretrained Transformer model directly fine-tuned on single-cell drug response data, without relying on bulk-to-single-cell transfer learning, for predicting single-cell drug reaction.

### Feature extraction from RNA-seq LLM and comparison model setting

In this study, the pre-trained single-cell expression models, scFoundation and Geneformer, serve as the foundational models for extracting bulk or single-cell feature embeddings that can be used for fine-tuning. The resulting feature embedding matrix $X$ should be $X\in{\mathbb{R}}^{N\times F}$, where $N$ represents the number of cell lines in bulk data or the number of cells in single-cell data, and $F$ represents the feature embedding dimension.

ScFoundation serves as a foundational model in the single-cell domain, utilizing the xTrimoGene [28] architecture with 100 million parameters. It has been pre-trained on a dataset of over 5 million single-cell expression profiles. The large model scale and extensive pretraining depth enable scFoundation to capture the intrinsic information of gene expression more effectively in the extracted features. In this experiment, feature embeddings from scFoundation are specifically extracted using max-mean pooling embedding for all genes and are concatenated with the embeddings of token T and S (indicators representing the total gene expression count), resulting in a feature embedding dimension of 3072.

Geneformer, another advanced foundational model in the single-cell domain, employs a BERT (Bidirectional Encoder Representations from Transformers) architecture [29], utilizing masked pre-training to capture gene interactions and relationships in expression profile data. In this experiment, feature embeddings from Geneformer are specifically extracted from the model’s penultimate layer as the extracted feature. Different versions of Geneformer (gf-6 L-30 M-original, gf-12 L-30 M-i2048) produce feature dimensions of 256 and 512, respectively.

Additionally, scFoundation supports a maximum fixed vocabulary of 19 264 genes, while Geneformer (6 L and 12 L variants) supports a fixed vocabulary of 25 426 genes. For each input, Geneformer only the top 2048 highly expressed genes are selected as gene tokens. Both scFoundation and Geneformer, along with comparative models like scDEAL, scFoundation-SCAD, and DrugFormer incorporate complex data preprocessing and gene matching methods, detailed in the Supplementary Methods.

### Res-VAE construction and training methods

The Feature Reconstruction Module aims to reconstruct features and obtain their lower-dimensional latent representation vector $z$ via variational inference. This module consists of a VAE model with residual connections, taking the feature embedding matrix $X$ extracted by the LLM as input and outputting the reconstructed ${X}^{\prime }$, which matches the dimensionality of $X$. The VAE is pre-trained on the PanglaoDB dataset using 10-fold cross-validation, ensuring a comprehensive evaluation of the model’s loss.

**Figure 2:**
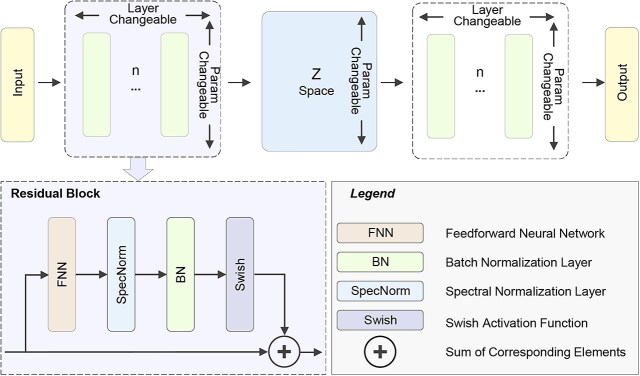
Hyperparameter tuning in the network of res-VAE model. Alt text: Schematic diagram of the res-VAE model architecture with tunable hyperparameters. The model consists of an encoder, latent space (Z space), and decoder, where the number of layers and parameters are configurable. A zoomed-in view of the residual block shows a feedforward neural network (FNN) followed by spectral normalization (SpecNorm), batch normalization (BN), and a swish activation function. Skip connections sum the input and output of the block. A legend explains the components and symbols used.

The Res-VAE model is designed with a fully connected Encoder-Decoder module, connected through a variational inference layer positioned in the middle. The Encoder-Decoder is composed of specially designed Residual Blocks (Fig. 2), with each Residual Block layer including a fully connected layer, spectral normalization layer (spectral regularization), batch normalization layer, Swish activation function [30], and a residual connection block [31]. The Swish function, compared to the traditional ReLU (Rectified Linear Unit) function, enables the VAE model to achieve better training and fitting performance, ultimately enhancing the model’s generalization ability [30]. The formula for the Swish activation function is as follows:


(1) \begin{equation*} Swish(x)=\frac{x}{1+{e}^{-\alpha x}} \end{equation*}

where $\alpha$ is a trainable parameter.

The residual connection block consists of a batch normalization layer and an adaptive dimensionality matching layer for adjacent layers. When the dimensions of adjacent layers match, the block acts as an identity mapping layer; when adjacent layers differ, it functions as a fully connected layer without an activation function.

The variational inference layer follows the $\beta$-VAE design [17, 32], using a single fully connected network representing the mean $u$ and another single fully connected network representing the log variance $\mathit{\log}\_\mathit{\operatorname{var}}$, approximating the latent representation vector $z$ with the following formula:


(2) \begin{equation*} Z=u+\varepsilon \cdot \exp \left(\frac{1}{2}\cdot \log \_\mathit{\operatorname{var}}\right) \end{equation*}

Where $\mathrm{\varepsilon}$ is sampled from a standard normal distribution of $\exp \left(\frac{1}{2}\cdotp \mathit{\log}\_\mathit{\operatorname{var}}\right)$, this is named as Reparameterization Trick.

Additionally, the Res-VAE is trained by optimizing a loss function that combines the reconstruction loss (REC) for ${X}^{\prime }$ and the Kullback–Leibler divergence (KLD) between the prior distribution $p(Z)$ and the approximate posterior $q(Z)$. The total loss function is given by:


(3) \begin{equation*} {\mathcal{L}}_{REC}=\frac{1}{N}\sum_{i=1}^N{\left\Vert{x}_i-{\hat{x}}_i\right\Vert}^2 \end{equation*}

(4) \begin{equation*} {\mathcal{L}}_{KLD}=-\frac{1}{2N}\sum_{i=1}^N\sum_{j=1}^d\left(1+\log \left({\sigma}_j^2\right)-{\mu}_j^2-{\sigma}_j^2\right) \end{equation*}

(5) \begin{equation*} {\mathcal{L}}_{total}=\beta \cdot{\mathcal{L}}_{REC}+{\mathcal{L}}_{KLD} \end{equation*}

where ${x}_i$ is the feature of the $i$th cell in the matrix $X$, and ${\hat{x}}_i$ is the feature of the $i$th cell in the matrix ${X}^{\prime }$. $d$ is the dimensionality of the latent representation vector $z$, ${\mu}_j$ is the mean of the $j$th latent variable. ${\sigma}_j$ is the standard deviation of the $j$th latent variable. $\beta$ is a hyperparameter controlling the trade-off between the REC and KLD to mitigate posterior probability collapse. It is set to 1000 in this experiment.

*Optuna* (Version: 3.6.1) [33] is deployed to search for the optimal hyperparameters of the Res-VAE model including the latent dimension of $z$, the dimension of each Residual Block Layer, the number of Residual blocks, and the learning rate $lr$. Specifically, VAE is pretraining under 5-fold cross-learning, and TPE (Tree-structured Parzen Estimator) is used to evaluate the best hyperparameter of minimum mean ${\mathcal{L}}_{\mathrm{total}}$ of five-fold in the validation set.

In the scATD-gf model, spectral regularization is applied to each learnable parameter to constrain the network’s Laplacian constant, which is critical for the stable training of VAEs [27]. In this experiment, incorporating spectral regularization helps prevent numerical overflow in the loss function due to unreasonable hyperparameter settings during the initial phases of Optuna optimization when pretraining scATD-gf.

### Knowledge distillation from res-VAE

scATD-sf-dist also adopts Res-VAE as its backbone (with change in hyperparameters), and its input is the gene expression profile ${X}_{exp}\in{\mathbb{R}}^{N\times G}$, reconstruct ${X_{exp}}^{\prime }$ as output. In this context, $N$ represents the number of cell lines in bulk RNA-seq data or the number of cells in single-cell RNA-seq data, and $G$ denotes the number of genes. The model is trained on the Panglao dataset by minimizing the REC loss, KLD loss, and an additional cosine similarity-based distillation (COD) loss. The COD loss measures the cosine distance between the latent representation vector ${f}_T$ from the scATD-sf model and the latent representation vector ${f}_S$ from the scATD-sf-dist for the same cell.


(6) \begin{equation*} {\mathcal{L}}_{COD}=1-\frac{f_{\mathrm{T}}\cdot{f}_S}{\left\Vert{f}_T\right\Vert \left\Vert{f}_S\right\Vert } \end{equation*}

The total loss function is:


(7) \begin{equation*} {\mathcal{L}}_{total}=\beta \cdot{\mathcal{L}}_{REC}+{\mathcal{L}}_{KLD}+\theta \cdot{\mathcal{L}}_{COD} \end{equation*}

where $\theta$ is a hyperparameter parameter to trade off distillation and reconstruction. $\beta$ is a hyperparameter with a similar purpose to Res-VAE and is set to 1000 in this experiment. $\gamma$ and other hyperparameters, including the number of network layers, were optimized using Optuna. The data partitioning for the training and validation sets follows the same 10-fold cross-validation approach as used for the scATD-sf and scATD-gf models.

### Domain adaptive transfer and drug reaction prediction

The domain adaptive transfer module is designed based on the pre-trained Res-VAE and the adaptive transfer block. The Encoder and variational inference layer of Res-VAE served as the stem network with fixed parameters inherent from the pretraining stage to accept bulk data as input, then add an additional Multilayer Perceptron (MLP) layer as a classification block for drug reaction prediction. Specifically, the stem network and classification block are conducting supervised learning on drug response labels in bulk data, the learning rate, model size, and other hyperparameters were fixed, with the number of epochs set to 150. During the first 130 epochs, only the parameters of the classification block were updated. In the final 20 epochs, updates to the stem network were also included. The classifier is configured with two output heads: one for drug sensitivity prediction and the other for resistance prediction (Fig. 1). The model’s classification loss is calculated using PyTorch’s built-in cross-entropy loss function (*torch.nn.CrossEntropyLoss*). Specifically, the model’s final output layer consists of two neurons for predicting drug sensitivity and drug resistance (which is why binary cross-entropy loss is not used). Bulk and single-cell datasets share the model, but the classification loss (cross-entropy loss) is computed only using labeled bulk data.

Concurrently with supervised learning at the bulk level, domain adaptation was achieved by aligning with single-cell drug features using either the AdaIN network (scATD-sf and scATD-gf) or Maximum Mean Discrepancy (MMD) loss (scATD-sf-dist) (Fig. 1). This alignment enabled the model to learn the relationship between single-cell features and drug sensitivity based on supervised labels from bulk data. After training, the network can directly accept scRNA-seq data as input to predict drug sensitivity.

In the scATD-sf or scATD-gf model, the AdaIN network sequentially performs phased style transfers to achieve bulk data feature transfer to single-cell data feature (the fixed Panglao data) during model training and subsequently from single-cell data feature (individual patient single-cell RNA-seq data) to bulk data feature during model inference (Fig. 1). The AdaIN formula is as follows:


(8) \begin{equation*} AdaIN\left(x,y\right)=\sigma (y)\left(\frac{x-\mu (x)}{\sigma (x)}\right)+\mu (y) \end{equation*}

where $x$ represents the features $X$ extracted by LLM from the source domain. When transferring bulk RNA-seq data to single-cell RNA-seq, $x$ is ${X}_{Bulk}$. While transferring single-cell data to bulk data, $x$ is ${X}_{sc}$. Similarly, $y$ represents the target domain features, which correspond to ${X}_{sc}$ in the case of bulk to single-cell transfer and ${X}_{bulk}$ is for single-cell to bulk transfer. Here, $\mu$ and $\sigma$ denote the mean and variance of the features, respectively.

Furthermore, the distillation model employs MMD loss to measure the statistical differences between the two latent representations encoded by the Res-VAE: bulk latent feature and single-cell latent feature. During training, the total loss is obtained by summing the cross-entropy loss and the MMD loss. By minimizing the MMD loss during model training, the transfer learning objective is achieved. The MMD loss function is defined as follows:


(9) \begin{align*} {MMD}^2\left({\mathcal{D}}_{s,}{\mathcal{D}}_t\right)=&\frac{1}{n_s^2}\sum_{i=1}^{n_s}\sum_{j=1}^{n_s}K\left({x}_i,{x}_j\right)+\frac{1}{n_t^2}\sum_{i=1}^{n_t}\sum_{j=1}^{n_t}K\left({y}_i,{y}_j\right)\nonumber\\&-\frac{2}{n_s{n}_t}\sum_{i=1}^{n_s}\sum_{j=1}^{n_t}K\left({x}_i,{y}_j\right) \end{align*}

where the Gaussian kernel function $K\left(x,y\right)=\exp \left(-\gamma \parallel x-y{\parallel}^2\right)$  $\gamma$ is a learnable parameter controlling the kernel scale. ${n}_s$ and ${n}_t$ represent the number of samples in the source domain ${\mathcal{D}}_s$ and target domain ${\mathcal{D}}_t$, ${n}_s$ equal to the ${n}_t$ controlled by training batch size. ${x}_i,{x}_j\in{\mathcal{D}}_s$ represents sampling from source domain (bulk) latent Z embedding, ${y}_i,{y}_j\in{\mathcal{D}}_t$ represents sampling from target domain (single cell) latent Z embedding.

The reason for adopting MMD rather than AdaIN in scATD-sf-dist is largely due to the AdaIN performance relying on feature extraction from LLM. For scATD-sf-dist, which directly extracts features from raw RNA-seq data, integrating AdaIN may not be inherently suitable due to the high-dimensional and sparse characteristics of raw RNA-seq data. To address this limitation, the scATD-sf-dist model adopts MMD to conduct transfer learning.

In addition, we include details on model training, inference, and the Bi-Adain and MMD-based domain adaptation procedures in the Supplementary Methods.

### Data augmentation strategy and model evaluation methods

Given the imbalanced drug response labels in bulk data, the k-nearest neighbors oversampling strategy, part of the Synthetic Minority Over-sampling Technique (SMOTE) data augmentation algorithm [34],was adopted to generate synthetic samples for the minority class. The number of neighbors was set to the default value of 5. Subsequently, Tomek links were employed to remove instances from the majority class that formed Tomek links with minority class samples, thereby clarifying the boundary between the majority and minority classes. For processing details, the library of *imblearn* (version: 0.12.3) was deployed to implement this strategy. Specifically, the SMOTE algorithm was deployed in the ablation of the Res-VAE model, scDEAL, and single-cell supervised training model.

In addition to the SMOTE algorithm, the pretraining scRNA-seq VAE model was deployed to execute data augmentation. Compared to the SMOTE technique, which linearly interpolates between neighboring data points for data augmentation, VAE-based augmentation can sample around the center of input data following the latent distribution learned by the VAE model. Detailed steps for VAE-based augmentation are provided in the Supplementary Methods.

After VAE augmentation, the Tomek link is also used to clarify the boundary between the minority and majority classes. VAE-based augmentation is deployed in the scATD-sf, scATD-gf, and scATD-sf-dist.

For the downstream RNA-seq drug response model, we used Accuracy, Recall, Precision, F1-score, and the Matthews correlation coefficient (MCC) to evaluate the prediction performance and generalization of these models. We also draw the receiver operating characteristic curve (ROC) and the precision-recall curve (PRC) to evaluate model performance at different thresholds. We also use Uniform Manifold Approximation and Projection (UMAP) and silhouette score [35] to evaluate feature embeddings from LLMs or VAE latent spaces. Details of the evaluation experiment setting and methods are provided in the Supplementary Methods.

### Biomarker identification and survival analysis

The biomarker selection and survival analysis module can assess the contribution of features sourced from RNA-seq LLM embeddings to downstream drug response prediction models, then key features will be identified and selected as biomarkers for patients’ survival risk analysis. Similarly, the scATD-sf-dist model can utilize this method to identify crucial genes.

Feature attribution values are obtained using an expectation-based version of the Integrated Gradients (IG) algorithm [20, 36] (also named Gradient SHAP) on the sensitivity and resistance prediction heads of the model, quantifying the contribution of each feature to both sensitivity and resistance predictions separately.


(10) \begin{align*} {IG}_{\mathrm{sensitivity},i}\left(\mathrm{x}\right)=&\left({x}_i-{x}_i^{\prime}\right)\times{\mathrm{E}}_{{\mathrm{x}}^{\prime}\sim \mathrm{P}\left({\mathrm{x}}^{\prime}\right)}\nonumber\\&\left[{\int}_{\alpha =0}^1\frac{\partial{F}_{\mathrm{sensitivity}}\left({\mathrm{x}}^{\prime }+\alpha \left(\mathrm{x}-{\mathrm{x}}^{\prime}\right)\right)}{{\partial x}_i} d\alpha \right] \end{align*}

(11) \begin{align*} {IG}_{\mathrm{resistance},i}\left(\mathrm{x}\right)=&\left({x}_i-{x}_i^{\prime}\right)\times{\mathrm{E}}_{{\mathrm{x}}^{\prime}\sim \mathrm{P}\left({\mathrm{x}}^{\prime}\right)}\nonumber\\&\left[{\int}_{\alpha =0}^1\frac{\partial{F}_{\mathrm{resistance}}\left({\mathrm{x}}^{\prime }+\alpha \left(\mathrm{x}-{\mathrm{x}}^{\prime}\right)\right)}{{\partial x}_i} d\alpha \right] \end{align*}

where, ${F}_{\mathrm{sensitivity}}$ represent the model output function for the sensitivity prediction head and ${F}_{\mathrm{resistance}}$ represent the resistance prediction head. $x$ represents an input feature vector and ${x}^{\prime }$ represents a baseline input vector. In this experminet, ${x}^{\prime }$ is defined as the mean of $x$. The feature ${x}_i$ denotes the $i$-th feature in the input vector.

Then, features with high absolute contribution values will be seen as the key features (or genes). The key feature embedding or gene expression value will be used to construct a Cox proportional hazards model, and generate a survival curve. Based on the median risk score, patients will be stratified into High-risk and Low-risk groups, enabling a comparative analysis of survival outcomes between these groups (Fig. 1).

## Results

### Drug latent knowledge representations in single cell

**Figure 3:**
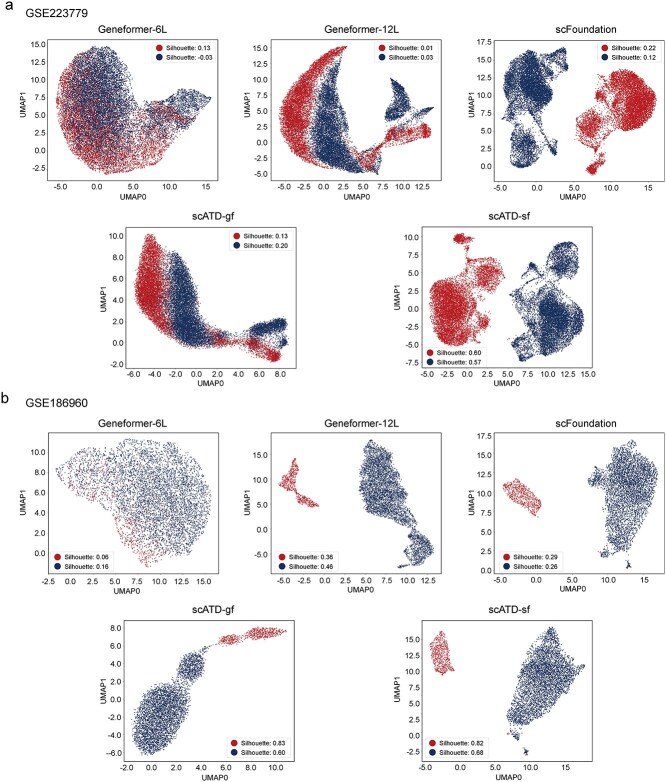
Feature latent representation visualization plot. (a). UMAP-2d plot of GSE223779. (b). UMAP-2d plot of GSE186960. Alt text: UMAP 2D projections of feature latent representations across two single-cell datasets (GSE223779, GSE186960). Each subplot represents a dataset, and corresponding model. Clusters indicate how well models separate cell populations (resistant versus sensitive) in the latent space.

In transfer learning, the quality of data representation influences the efficacy and interpretability of the prediction model [37]. Insights from in-context learning (ICL) suggest that the task-relevant representational knowledge acquired during the pre-training of LLMs influences downstream task performance [38]. To investigate the representational knowledge related to drug resistance of various LLMs and the scATD model, UMAP dimensionality reduction was applied to features extracted from LLMs of varying scales, including Geneformer (gf-6 L-30 M-original, gf-12 L-30 M-i2048) and scFoundation, across datasets GSE223779, GSE186960, and GSE163836 (Fig. 3a and b and Fig. S1a). Results indicate that scFoundation distinctly separates drug resistance and sensitivity clusters, whereas Geneformer (gf-12 L-30 M-i2048) achieves clear separation in GSE186960 and GSE163836 but is less distinct in GSE223779. Geneformer (gf-6 L-30 M-original) fails to form clear clusters in all three datasets. Clustering is suboptimal across GSE202234 and GSE149383 for all models (Fig. S1b and c). Silhouette scores (Table 2) confirm that scFoundation outperforms Geneformer on average, with the latter’s smaller model (gf-6 L-30 M-original) performing the worst. These findings highlight how model architecture and parameterization affect representation quality, with larger architectures offering better pre-existing knowledge. However, even domain-specific models like scFoundation and Geneformer show limited performance on certain datasets.

**Table 2:** Silhouette scores of sensitive and resistant groups across different model representation.

**Dataset**	**gf_6L_LLM**		**gf_12L_LLM**		**scATD-gf**		**scFoundation**		**scATD-sf**	
	**sensitive**	**resistant**	**sensitive**	**resistant**	**sensitive**	**resistant**	**sensitive**	**resistant**	**sensitive**	**resistant**
GSE186960	0.06	0.16	0.36	0.46	**0.83**	0.60	0.29	0.26	0.82	**0.68**
GSE163836	0.16	0.08	0.12	0.20	0.53	0.27	0.38	0.28	**0.78**	**0.63**
GSE108383_A375	0.23	**1.00**	0.08	**1.00**	0.43	0.88	0.06	0.11	**0.63**	0.74
GSE112274	0.09	0.07	0.19	0.07	0.48	0.15	0.42	0.27	**0.91**	**0.41**
GSE223779	0.13	−0.03	0.01	0.13	0.13	0.20	0.22	0.12	**0.60**	**0.57**
GSE108397	−0.01	0.07	−0.03	0.10	0.09	0.18	0.14	0.13	**0.54**	**0.60**
GSE117872_HN120	0.01	0.10	−0.03	0.07	−0.10	0.19	**0.02**	0.27	−0.05	**0.79**
GSE140440	−0.02	0.13	−0.06	0.08	−0.06	0.09	0.06	0.17	**0.24**	**0.34**
GSE111014	−0.09	0.15	0.02	0.09	0.09	0.13	0.03	0.10	**0.19**	**0.35**
GSE117872_HN137	0.02	0.09	0.00	0.04	−0.01	0.18	**0.09**	0.06	−0.01	**0.51**
GSE131984	0.13	−0.04	0.04	0.04	0.12	0.05	0.13	0.08	**0.22**	**0.24**
GSE108383_X451	0.00	0.01	0.00	0.01	0.03	−0.02	0.06	0.03	**0.16**	**0.06**
GSE149214	−0.05	0.10	−0.05	0.10	−0.09	**0.14**	−0.02	0.10	**0.05**	0.12
GSE149383	−0.04	0.07	−0.04	0.08	−0.08	**0.11**	−0.02	0.09	**0.05**	0.09
GSE169246	**0.02**	−0.01	0.01	0.02	−0.07	0.10	0.00	0.03	−0.07	**0.17**
GSE202234	0.16	−0.05	0.11	−0.04	**0.17**	**−0.04**	0.11	−0.05	0.17	−0.09
Mean	0.05	0.12	0.05	0.15	0.16	0.20	0.12	0.13	**0.33**	**0.39**

gf_6L_LLM represents feature representation of Geneformer (gf-6 L-30 M-original), gf_12L_LLM represents feature representation of Geneformer (gf-12 L-30 M-i2048), scATD-gf represents feature representation of scATD-gf latent Z layer, scFoundation represents feature representation of scFoundation and scATD-sf represents feature representation of scATD-gf latent Z layer. The bold values indicate the model with the highest Silhouette score for each dataset, representing the best clustering performance.

Furthermore, based on the scATD architecture, by training scATD-sf and scATD-gf, latent representations of LLM features are extracted, which theoretically provide superior feature information representation. For datasets GSE223779, GSE186960, and GSE163836, scATD-sf’cATD-sf representations of LLM features are extracted, whic’s clustering performance and achieve higher silhouette scores across sixteen datasets (Fig. 3a and b and Fig. S1a, Table 2). scATD-gf also forms sensitive-resistant cluster structures relatively well in these three datasets, with an average silhouette score higher than Geneformer features.

Additionally, scATD-sf and scATD-gf exhibit stable training and good loss fitting during reconstruction (Fig. S2). These results support that during the pre-training reconstruction phase in scATD, Res-VAE effectively captures latent information from LLMs. Considering that Res-VAE has much smaller parameters than LLMs, this could provide an advantage in training efficiency for subsequent drug response and knowledge distillation phase.

### Drug reaction prediction transfer from bulk to single cell RNA-seq

Typically, IC50 values are used to annotate bulk sequencing data from patient tissue samples, thereby determining the overall drug response of the patient [39]. For single-cell drug response profiling, untreated cells are classified as sensitive, while cells that endure drug exposure and survive are labeled as resistant. Predictive models for single-cell responses follow two main approaches (Table S1): supervised learning on labeled single-cell data (e.g. DrugFormer) or transfer learning from bulk data with domain adaptation to single-cell data (e.g. scDEAL, scFoundation-SCAD).

**Figure 4:**
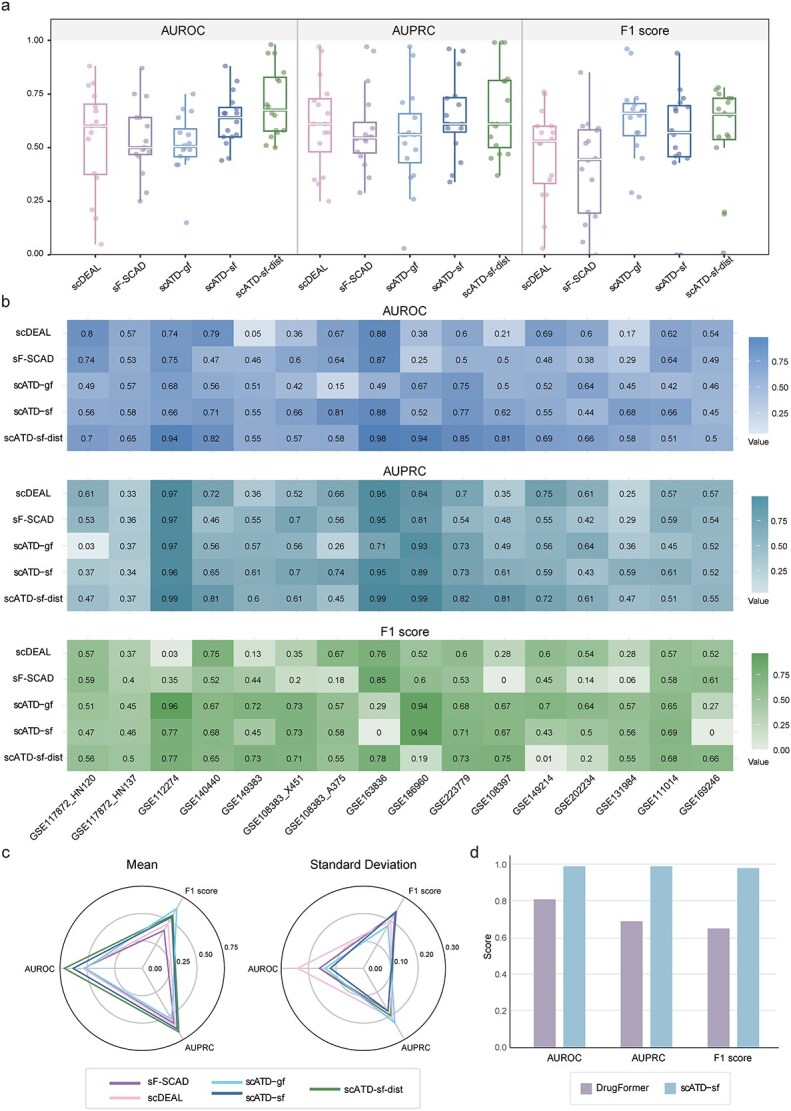
Evaluation results across sixteen single-cell independent datasets. (a). Boxplot of model prediction evaluation matrixes. (b). Heatmap of model prediction evaluation matrixes. (c). Radar plot of model prediction evaluation matrixes. (d). Mean model prediction of evaluation matrixes. Specifically, sF-SCAD is an abbreviation for scFoundation-SCAD. Alt text: Four-panel figure comparing model performance across 16 single-cell datasets. (a) Boxplots of evaluation metrics (e.g. AUROC, AUPRC) for multiple models. (b) Heatmap showing metric values across models and datasets, with higher scores in darker shades. (c) Radar plots illustrating model performance across evaluation metrics. (d) Bar chart of mean performance per model. scATD-sf has higher than DrugFormer.

The scATD framework employs a bidirectional AdaIN transfer learning strategy to train a total of 10 scATD-sf and 10 scATD-gf models for 10 drug-cancer types. These models allow adaptive inference on single-cell datasets that belong to the same drug-cancer type without additional parameter training, making them suitable for edge-computing scenarios (e.g. clinical deployment) and differing from previous studies (scDEAL and scFoundation-SCAD). Due to its nonparametric training nature and the fact that single-cell labels are not required, all single-cell datasets were treated as independent test sets. This transfer process aligns with the model’s deployment behavior in real-world applications. Compared to scDEAL and scFoundation-SCAD, scATD-sf achieved superior performance with higher average AUROC, AUPRC, and F1 scores across sixteen datasets and lower standard deviations. While scATD-gf showed slightly lower AUROC and AUPRC, it had higher F1 scores (Fig. 4a–c).

In particular, for five datasets reported by scDEAL (GSE117872_HN120, GSE117872_HN137, GSE112274, GSE140440, and GSE149383) [5], the scATD-sf model achieved higher AUROC values compared to both scDEAL and scFoundation-SCAD on the GSE117872_HN137 and GSE149383 datasets. Additionally, in two datasets reported by the SCAD model (GSE108383_X451 and GSE108383_A375) [4], the scATD-sf model outperformed scDEAL in AUROC values (Fig. 4b). These experimental results indicate that the scATD-sf model demonstrates relatively better performance compared to scDEAL and scFoundation-SCAD. On the other hand, scATD-gf generally performs worse than scATD-sf, which corresponds to its feature representation performance.

For scATD-sf-dist (distillation from scATD-sf), which adopts MMD for transfer learning, retraining is required for each single-cell dataset; however, it does not require labels for the single-cell data, and this transfer behavior aligns with the model’s deployment in real-world applications. It demonstrated higher predictive accuracy than scDEAL, with an average AUROC of 0.71 and AUPRC of 0.67. scATD-sf-dist outperformed scATD-sf in 12 out of 16 datasets (Fig. 4b), with significant improvements on datasets like GSE112274 and GSE186960. To ensure a fair comparison, data with AUROC <0.5 were removed. scATD-sf-dist still outperforms the comparison models, and both scATD-sf-dist and scATD-sf have more data with AUROC >0.5 (Table S2).

**Figure 5:**
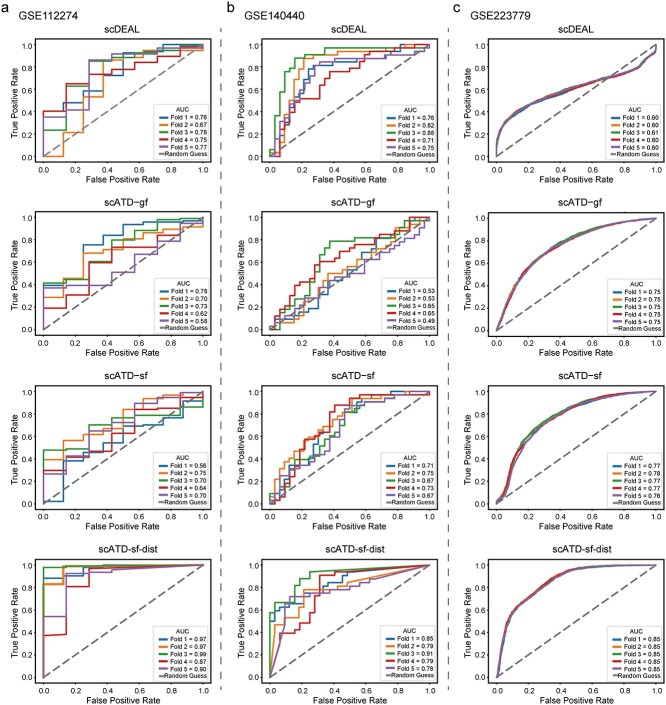
ROC plot of model five-fold inference. (a). The ROC curve of the GSE112274 dataset. (b). The ROC curve of the GSE140440 dataset. (c). The ROC curve of the GSE223779 dataset. Alt text: ROC curves across 5-fold cross-validation for three datasets (GSE112274, GSE140440, GSE223779) and four models (scDEAL, scATD-gf, scATD-sf, scATD-sf-dist).

To evaluate the robustness of model predictions across varying data distributions, model inference under five-fold partitioned data [17] is conducted on three single-cell datasets (Fig. 5). In the GSE112274 dataset, scATD-sf-dist achieved the highest average AUROC with minimal standard deviation and the highest average MCC among all models (Fig. 5a, Table S3). For the GSE140440 dataset, scATD-sf-dist achieved the highest AUROC and a slightly lower MCC (0.496) than scDEAL (0.5) but showed lower MCC variability, indicating greater stability (Fig. 5b, Table S3). On the GSE223779 dataset, scATD-sf-dist outperformed others in both average AUROC and MCC (Fig. 5c, Table S3), highlighting its predictive performance advantage.

On the other hand, since the DrugFormer model directly uses single-cell drug response labels for supervised training, we evaluated the performance of the scATD-sf model through 5-fold cross-validation (dividing the target single-cell data into training and testing datasets) to make a comparison. scATD-sf achieved near-perfect AUROC, AUPRC, and F1 scores across all sixteen test datasets, while DrugFormer showed lower performance, with an average AUROC of 0.76 and lower AUPRC and F1 scores (Fig. 4d, Table S4). These results are higher than the accuracy observed in transfer learning experiments.

However, due to the unique nature of single-cell label annotation methods (i.e. treating drug-treated surviving cells as resistant, untreated cells are considered sensitive) and the inherent variability among patient, evaluation in validation datasets may be not generalized to another patients. In comparison, domain-adaptive transfer learning methods, such as Bi-AdaIN and MMD, due to their algorithmic design, provide evaluations on the target datasets that more closely reflect real-world generalization scenarios. To fairly evaluate generalization, scATD-sf (supervised learning in single cell label) was tested on independent datasets involving the same drug and disease types. High accuracy was observed for datasets similar to the training set (e.g. GSE108397 and GSE108383_X451 from 451Lu cells), In other cases, there was a substantial decrease in model performance (Table 3).

**Table 3:** Supervised model inference results in independent single-cell data.

**Group**	**Pretraining dataset**	**Inference dataset**	**AUROC**	**MCC**	**F1 Score**	**Recall**	**Accuracy**	**Precision**	**AUPRC**
1	GSE108383_X451	GSE108383_A375	0.34	0	0.57	1	0.4	0.4	0.32
		GSE108397	0.95	0.78	0.88	0.81	0.88	0.95	0.95
	GSE108383_A375	GSE108383_X451	0.56	−0.04	0.1	0.05	0.43	0.5	0.62
		GSE108397	0.18	0	0	0	0.5	0	0.34
	GSE108397	GSE108383_X451	0.84	0.54	0.7	0.55	0.73	0.95	0.9
		GSE108383_A375	0.5	0	0.57	1	0.4	0.4	0.4
2	GSE117872_HN120	GSE117872_HN137	0.62	0.1	0.4	0.38	0.62	0.36	0.35
	GSE117872_HN137	GSE117872_HN120	0.56	−0.22	0.02	0.02	0.56	0.05	0.34
3	GSE112274	GSE202234	0.54	0.04	0.35	0.27	0.53	0.51	0.52
	GSE202234	GSE112274	0.45	0	0	0	0.07	0	0.92
4	GSE163836	GSE169246	0.47	−0.05	0.68	0.91	0.53	0.54	0.55
	GSE169246	GSE163836	0.24	−0.04	0.84	0.99	0.73	0.73	0.61

In summary, these findings underscore the challenge of applying supervised fine-tuning (SFT) for pre-trained models on single cell datasets, primarily due to the substantial pathological differences among patients. The degree to which such heterogeneity affects model generalization remains to be determined. Given that bulk cohorts contain drug response information from numerous patients, combining this data with unsupervised adaptive transfer learning may facilitate the model’s ability to learn more fundamental single-cell features linked to drug response patterns, emphasizing the importance of scATD-related transfer learning methods in improving model generalization across diverse patient conditions.

### Ablation and inference latency experiments

**Figure 6:**
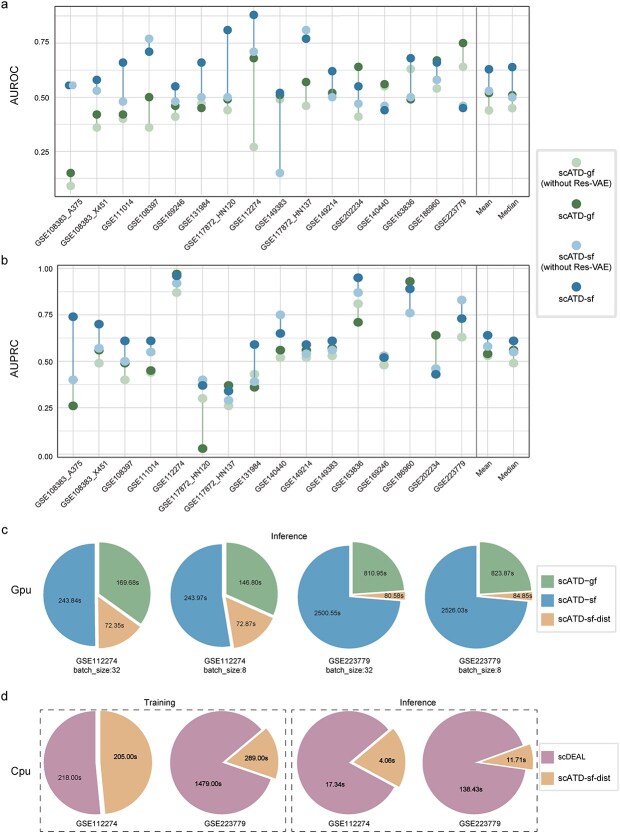
Results of ablation and inference latency experiment. (a). AUROC results of ablation experiments across 16 single-cell datasets. (b). PRROC results of ablation experiments across 16 single-cell datasets. (c). Time consumption of inference experiments based on GPU. (d). Time consumption of training and inference experiments based on CPU. Alt text: Composite figure with four panels showing ablation performance and latency. (a) Dot plot of AUROC for four models (geneformer light green, scATD -gf dark green, scFoundation light blue, scATD-sf dark blue) across 16 single-cell datasets, with vertical lines isolation for mean and median. (b) Same layout for AUPRC values. (c) Four GPU inference time pie charts for GSE112274 and GSE223779 at batch sizes 32 and 8, comparing scATD -gf, scATD-sf, and scATD-sf-dist slices. (d) Four CPU time pie charts grouped into training (GSE112274, GSE223779) and inference (GSE112274, GSE223779), comparing scDEAL (purple) and scATD-sf-dist (orange).

To evaluate the impact of critical components in scATD, ablation experiments were conducted on all single-cell drug sensitivity datasets (Fig. 6a and b and Table S5). Results showed that integrating Res-VAE improved performance. scATD-gf and scATD-sf models achieved higher AUROC and AUPRC scores on average compared to directly using GeneFormer or scFoundation features without the Res-VAE module. This means that when scATD-sf and scATD-gf are omitted, LLM embeddings are directly used with Bi-AdaIN for prediction. For AUROC, scATD-gf outperformed gf in 14 of 16 datasets (mean AUROC: 0.52 versus 0.44), and scATD-sf outperformed sf in 11 datasets (mean AUROC: 0.63 versus 0.53). Similar improvements were observed in AUPRC values, with scATD-gf and scATD-sf showing higher averages and reduced variation across datasets. These findings highlight Res-VAE’s ability to enhance representational performance.

Additionally, experiments were conducted to validate the impact of the Bi-AdaIN module on model performance. When removing the Bi-AdaIN transfer module from the scATD-gf and scATD-sf models, the performance of the scATD-sf model decreased across most datasets, with notable declines in some cases (Fig. S3 and Table S5), though slight improvements in AUROC and AUPRC accuracies were observed for datasets GSE140440 and GSE169246. On the other hand, the scATD-gf model exhibited varying degrees of reduction in AUROC accuracy across 12 of 16 datasets and a decrease in AUPRC accuracy across 11 of 16 datasets (Fig. S3 and Table S5). Overall, the Bi-AdaIN transfer module enhances predictive accuracy when transferring models trained on bulk data to single-cell predictions. Combining the experimental results from Chapter 3.2 (Table 3), where the scATD-sf model without Bi-AdaIN was directly trained on single-cell data and subsequently applied to other single-cell datasets, the results suggest that while the pretraining-finetuning strategy of large foundation models is an effective transfer learning approach, transferring knowledge from bulk RNA-seq data remains essential for accurately predicting single-cell drug reaction.

In clinical applications, rapid disease diagnosis and treatment response are crucial. Inference latency experiments were conducted for scATD models (scATD-sf, scATD-gf, scATD-sf-dist) and scDEAL on NVIDIA A100 (80G) using GSE112274 (small data) and GSE223779 (large data) datasets (Fig. 6c). Among these, the scATD-sf-dist model had the shortest inference times, while scATD-sf was the slowest. This was due to scATD-sf’s higher feature extraction time (scFoundation) compared to scATD-gf (Geneformer), and scATD-sf-dist’s direct feature extraction from raw gene profiles contributed to its speed advantage. The latency difference was more pronounced with larger datasets, highlighting significant inference delays in large models for real-world applications.

Additionally, scATD-sf-dist and scDEAL were compared for training and inference time on a CPU (See Table S6 for experimental settings). On the smaller GSE112274 dataset, scATD-sf-dist’s training time was slightly shorter than scDEAL’s, while on the larger GSE223779 dataset, scATD-sf-dist significantly reduced training time to 289 s, five times faster than scDEAL’s 1479 s (Fig. 6d). For inference, scATD-sf-dist completed in 4.06 s on GSE112274 compared to scDEAL’s 17.34 s and 11.71 s on GSE223779, over 11 times faster than scDEAL’s 138.43 s (Fig. 6d). These results highlight scATD-sf-dist’s superior efficiency in both training and inference.

Although the speed of scATD-sf is slower than smaller models, its characteristic of not requiring retraining of model parameters enables rapid adaptation to different patients with the same cancer-drug combination. Hence, both models in scATD address the need for high-throughput and accurate predictions in single-cell cancer drug response prediction.

### Analysis of AdaIN’s role and its generalization to RECIST label prediction

Further analysis of the role of AdaIN in scATD was performed using dataset GSE112274 as an example. Specifically, the KL divergence was calculated between each dimension of the latent Z embedding (from the Res-VAE module within scATD-sf) using bulk data versus single-cell data (GSE112274) before and after the application of Bi-AdaIN transformation. The method for calculating the KL divergence followed the previously described KL loss formulation, and the Bi-AdaIN transformation followed the procedures described earlier. Results showed that before Bi-AdaIN transfer, the bulk and GSE112274 single-cell data exhibited larger average KL divergence and greater standard deviation across feature dimensions, indicating larger distributional discrepancies. In contrast, following Bi-AdaIN transfer, the distributions of the two datasets became substantially more aligned (Table S7). Visualizing the numerical distributions of the five features with the smallest post-transfer KL divergence demonstrated that AdaIN primarily functions by aligning the feature distributions between bulk and single-cell datasets (Fig. S4).

Considering the origin of the labels, the drug response annotations for bulk data originate from IC50/AUC-based drug sensitivity assays conducted on cancer cell lines, whereas single-cell drug response labels are commonly inferred from treatment conditions and cell survival status [5]. Nevertheless, both label types fundamentally originate from in vitro cellular experiments. Therefore, although one represents patient-level and the other indicates cellular-level, the prediction tasks are closely related, as both derive from similar in vitro experimental setups. Thus, after applying the AdaIN transfer, the model can effectively generalize predictions from bulk to single-cell data. A critical question arises regarding tasks with greater inherent differences, such as drug response labels directly derived from clinical RECIST criteria. RECIST is the gold-standard clinical evaluation method [40], assessing patient tumor responses based on therapeutic outcomes including tumor size reduction and clinical improvement. Investigating whether models trained on IC50-labeled bulk data can generalize to RECIST-defined labels through AdaIN or other transfer learning methods (Table S1) would provide valuable insights into the adaptability and effectiveness of AdaIN in diverse predictive scenarios.

Following the approach proposed by Ogunleye *et al.* [41, 42], the TCGA BRCA dataset of patients treated with Paclitaxel (designated as the BRCA_RECIST dataset) and the TCGA PAAD dataset of patients treated with Gemcitabine (PAAD_RECIST dataset) were selected as experimental datasets. Results indicated that irrespective of whether AdaIN or other transfer learning strategies were applied, both scATD and baseline models trained on IC50-based bulk drug response data struggled to achieve acceptable prediction performance on RECIST-defined clinical drug response labels (Table 4). Notably, like the previous 16 single-cell datasets (GSE datasets) experiments, transfer learning models only utilized the RNA-seq data from the target domain without accessing their corresponding labels. Hence, these datasets should be regarded as an independent validation set, as its labels were neither used nor accessible. Although, AdaIN transfer reduces the average KL divergence between bulk and TCGA datasets features (using the latent Z embedding from the scATD-sf Res-VAE module), indicating partial feature alignment (Table S7). However, this alignment did not translate into model performance improvements, suggesting that feature alignment alone may not be sufficient when the type of source and target domain labels differ significantly.

**Table 4:** Cross-domain RECIST-base label prediction with models trained on IC50 response labels.

**Model**	**BRCA_Paclitaxel**				**PAAD_Gemcitabine**			
	**AUROC**	**AUPRC**	**F1 Score**	**MCC**	**AUROC**	**AUPRC**	**F1 Score**	**MCC**
scATD-sf (Non_adain)	**0.76**	**0.48**	0	0	0.57	**0.67**	0	0
scATD-gf (Non_adain)	0.36	0.15	0	−0.22	**0.59**	0.68	0.20	**0.23**
scATD-sf (with_adain)	0.60	0.25	0.29	**0.17**	0.45	0.51	0.48	−0.04
scATD-gf (with_adain)	0.49	0.19	0.26	−0.01	0.42	0.51	**0.53**	−0.12
scATD-sf-dist	0.58	0.22	**0.31**	0.13	0.53	0.64	0.15	0.20
scfoundation- SCAD	0.64	0.38	0	0	0.59	0.64	0.29	0.03
scDEAL	0.42	0.20	0.29	0.18	0.54	0.61	0	0

Bold values represent the highest prediction performance for each evaluation setting, highlighting the best-performing model in each case.

A plausible explanation is that, algorithmically, methods such as AdaIN, MMD (used in scATD-sf-dist), and DANN (utilized in scfoundation-scad) fundamentally rely on transferring the source domain’s label-feature mapping relationships to unlabeled target-domain data. Theoretically, such transfer learning methods are most effective when the task labels of the source and target domains are closely related. Clearly, drug response labels defined by RECIST criteria and IC50-based assays do not fulfill this criterion. To further validate this hypothesis, an additional experiment was conducted: from TCGA BRCA, patient drug responses to Docetaxel were selected (excluding patients overlapping with the Paclitaxel drug used), resulting in a set consisting solely of drug response positive labels (RECIST-defined). Furthermore, two additional BRCA patients treated with Tamoxifen (non-response) who did not overlap with the other drug treatments were randomly included, resulting in a total of 24 patients. Docetaxel was chosen primarily because it belongs to the same taxane class as Paclitaxel and sufficient available records within TCGA-BRCA. We training the scATD-sf model on this RECIST-defined dataset (using RECIST-defined label), combined with AdaIN transfer, demonstrating substantial improvement in predictive performance on the BRCA_RECIST dataset (Paclitaxel response), achieving an AUROC of 0.81, AUPRC of 0.39, F1-score of 0.5, and MCC of 0.45. This indicates that AdaIN-based transfer learning can achieve strong predictive performance when the source and target domains share closely related label definitions. Additionally, it should be noted that for drug sensitivity prediction using RECIST-defined labels, a preferable practice is to train the models directly on RECIST labels, as demonstrated in work by Ogunleye *et al.* [41, 42].

### scATD for biomarker identification at patient level

In clinical applications, identifying biomarkers is vital for understanding cancer mechanisms and assessing the potential of prediction models in clinical applications [13, 43]. The IG algorithm is applied in the scATD framework with the scATD-sf model to compute feature attribution values, assessing the contribution of LLM features to model predictions at both population (bulk) and single-cell levels. For scATD-sf-dist, IG directly links gene expression to predictions, identifying key genes as biomarkers [44]. Notably, IG separately computes attribution for sensitivity and resistance prediction heads, which may result in asymmetric. Hence, the integrated results from both heads are compared for enhanced interpretability.

**Figure 7:**
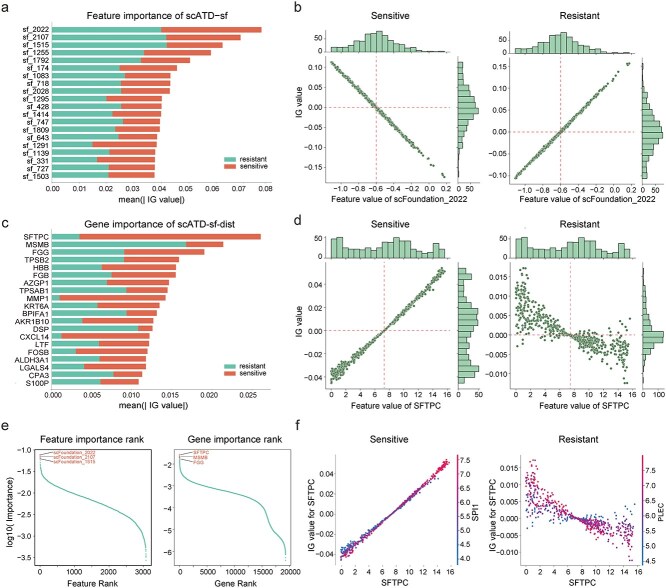
Feature importance and contribution plot. (a). Feature overall importance plot. Absolute values of feature attributions calculated using integrated gradients (IG) (b). Histogram plot of feature attribution values for the two prediction heads. The x-axis represents feature values, and the y-axis shows attribution values indicating whether each feature contributes positively or negatively to the model prediction. (c). Gene overall importance plot. (d). Histogram plot of gene attribution value for the two prediction heads of the model. The x-axis represents gene expression value, the y-axis represents gene attribution value. (e). Feature or gene importance rank distribution plot. (f). Dependence plot of target gene. The x-axis represents the value of target gene expression value, the left y-axis represents the attribution value of the target gene, while the right y-axis represents the expression value of the interaction gene. Alt text: Six-panel figure: (a) Bar chart of the top 20 features’ mean absolute integrated gradients (IG) attributions, separately shown for the resistant and sensitive prediction heads. (b) Scatter plots with marginal histograms of feature scFoundation_2022 value versus IG attribution for the sensitive and resistant heads. (c–d) Same as (a–b) but for the top 20 genes and for SFTPC expression. (e) Scatter plots of log₁₀(importance) versus rank for features and genes. (f) Dependence plots of SFTPC IG attribution versus expression, colored by interacting-gene expression.

Using the Gefitinib drug-trained scATD-sf model as an example to identify biomarkers in the TCGA LUAD cohort dataset, the feature “scFoundation_2022” (2021st dimension of the scFoundation embedding) showed the overall highest importance (Fig. 7a and e). In the resistance head, when a patient carries low feature values below (approximately −0.6), the effect of this feature is interpreted as promoting the model’s predicted sensitivity to Gefitinib, while higher values have the opposite effect, the sensitivity head also supports a similar conclusion, reflecting a good level of symmetry (Fig. 7b). Given “scFoundation_2022” strong ability to distinguish Gefitinib resistance risk among patients, along with its largest impact and high contribution ration to model predictions, it can serve as a potential biomarker for predicting Gefitinib resistance in LUAD patients.

In the scATD-sf-dist model trained on the same dataset, *SFTPC*, *MSMB*, and *FGG* were identified as the top 3 overall important genes (Fig. 7c and e). In the resistance prediction head, their rankings were 88/19264, 1/19264, and 5/19264, respectively, and for the sensitivity head, 1/19264, 38/19264, and 4/19264. *FGG* showed strong symmetry. For patients who carry *FGG* with high expression, the gene effect is interpreted as promoting the model’s predicted sensitivity to Gefitinib (Fig. S5a and c). *MSMB* exhibited asymmetry (Fig. S5b). *SFTPC*’s influence was clear in the sensitivity head, but was complex in the resistance head (Fig. 7d). Based on the SHAP package [14], we also observed *PLEC* and *SPI1* as key interacting genes for *SFTPC* in the resistance and sensitivity heads, respectively, highlighting distinct interaction patterns (Fig. 7f).

Medical literature supports *SFTPC*’s role in enhancing the anti-cancer immune environment [45], with low expression linked to poor LUAD prognosis [46]. While direct evidence linking *SFTPC* expression levels to Gefitinib efficacy in LUAD is lacking, higher *SFTPC* expression is associated with better LUAD outcomes. This suggests a lower potential risk of Gefitinib resistance, aligning with the model’s interpretability analysis results.

Furthermore, Gene Ontology (GO) enrichment analysis of the top 10 key genes revealed potential biological pathways associated with LUAD development, including immune-related processes, cell adhesion, platelet aggregation, and protein activation cascades (Fig. S5d). Notably, four representative genes—*AREG*, *EREG*, *VEGFA* and *CD274* (PD-L1)—critical for both Gefitinib resistance and LUAD progression [47–49], were ranked with importance scores of 542/19264 (top 2.81%), 618/19264 (top 3.21%), 1630/19264 (top 8.46%), and 3315/19264 (top 17.21%), respectively. *AREG*, *EREG*, and *VEGFA* are angiogenesis-related and often overexpressed in tumor cells. In contrast, *CD274*, a key immune checkpoint, may show low expression in certain tumors, complicating the signaling of its role in drug resistance. Although the scATD-sf-dist model is trained on gene expression data, it can also capture signals from *CD274*, even in patient cohorts not specifically designed for drug resistance experiments. This further demonstrates that even using IG-based gene selection alone, scATD-sf-dist can identify key genes established by ground truth research.

**Figure 8:**
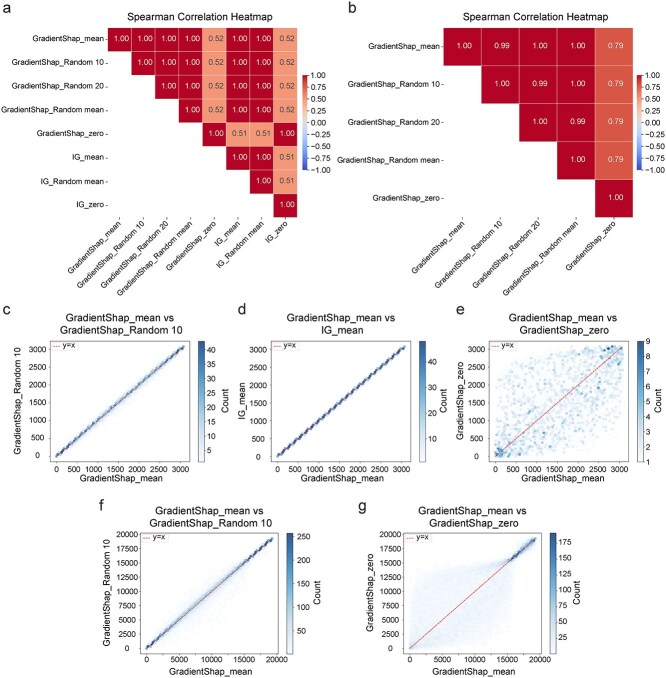
Rank robustness analysis across different baseline model in TCGA_LUAD. (a). Spearman coloration plot of feature importance rank in scATD-sf. Gradient SHAP is an expectation-based version of the integrated gradients (IG). Mean represent the mean of all sample’s feature as baseline. Random10, random 20 represent randomly selecting 10 or 20 samples from the dataset as the baseline (multiple baselines). Random mean represents the average of 10 randomly selected samples from the dataset as the baseline. Zero represents a zero baseline. (b). Spearman coloration plot of gene importance rank in scATD-sf-dist. Mean represent the mean of sample’s gene expression value as baseline. (c), (d), (e) correspond to the results of feature importance rank distribution from scATD-sf. (f) and (g) correspond to the results of gene importance rank from scATD-sf-dist. Alt text: Composite figure of seven panels comparing attribution methods. (a) Spearman correlation heatmap among eight methods-baseline composition (coefficients 0.52–1.00). (b) Spearman correlation heatmap for five baseline (0.79–1.00). (c–e) Density scatterplots of feature importance rank distribution: GradientShapmean versus (GradientShapRandom10, Igmean, GradientShapzero), (g–f) density scatterplots of gene importance rank distribution: GradientShapmean versus (GradientShapRandom10, GradientShapzero). Most clustered along the diagonal except GradientShapmean versus GradientShapzero scatter at extended range; each includes a dashed y = x reference line and color-coded point density.

Additionally, experiments incorporating different interpretation techniques and multiple baseline choices (Fig. 8, Fig. S6) demonstrate that the zero baseline (default setting in Captum package and displayed in scDEAL) poses potential risks in interpretation analysis. Under this setting, all genes, regardless of expression level, are interpreted as producing a unidirectional effect on drug resistance (Fig. S6a–d). This issue may arise from the incorrect assumption that background gene expression is zero. In contrast, scATD uses the mean of feature or gene expression value as the baseline (Fig. 8), showing high correlation (close to 1) with importance rankings derived from multiple baselines (except zero baseline), and demostrate opposite-direction effect on drug resistance based on different gene expression value (Fig. S6  e and f), making scATD interpretation more reasonable.

In summary, the key features and genes identified in the patient cohort level by the scATD model are not only clinically relevant but also provide deep interpretable insights. Making it a potential biomarker to support clinical analysis.

### Biomarker identification at single cell level

scATD enables single-cell resolution interpretations for each patient. Taking prostate cancer patients undergoing Docetaxel treatment as examples, six single-cell sequencing datasets in the GSE137829 were analyzed. Key features and genes were identified using the scATD-sf and scATD-sf-dist models. For patient 1, the top three features were scFoundation_90, scFoundation_207, and scFoundation_2511, while the top three genes were *RPS6*, *RPLP1*, and *RPL41* (details for others in Table S8).

**Figure 9:**
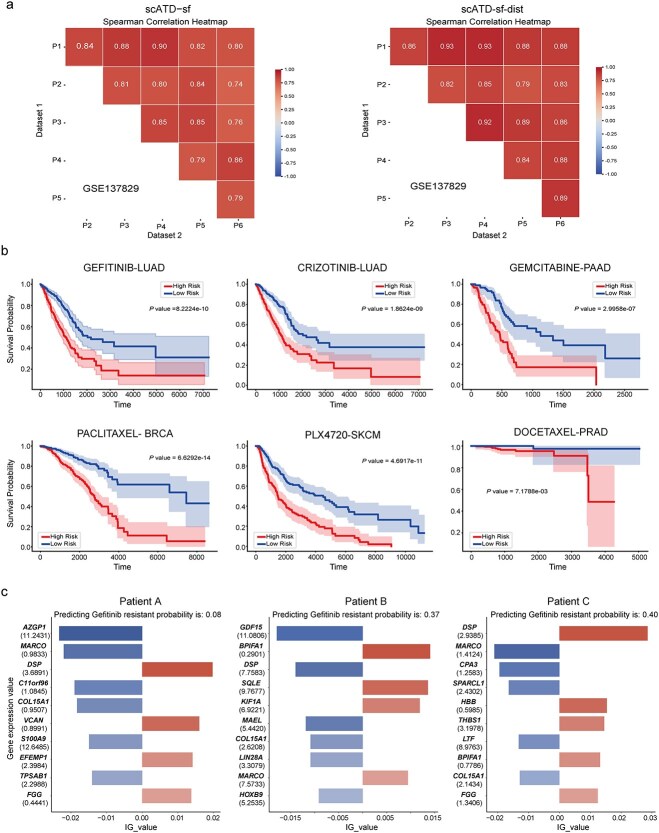
Rank perturbation across six patient and survival prognosis analysis. (a). Spearman coloration plot of feature importance rank. The left evaluates scATD-sf feature importance rank similarity, the right evaluates the scATD-sf-dist gene importance rank similarity. (b). Survival plot of six datasets across five patient cohort. (c). Probability of Gefitinib resistance in OS-0-Max3 in TCGA_LUAD (Gefitinib-LUAD). Alt text: Composite figure with three panels: (a) two spearman correlation heatmaps comparing latent representations across six cancer datasets (P1–P6) for VAE-sf (left) and Dist-VAE (right), with correlation coefficients ranging from 0.74 to 0.93. (b) Six Kaplan–Meier survival curves showing high-risk versus low-risk patient groups under different gene group, with statistic test: Gefitinib-LUAD (*P* = 8.22 × 10^−10^), Crizotinib-LUAD (*P* = 1.86 × 10^−9^), gemcitabine-PAAD (*P* = 2.995 × 10^−7^), paclitaxel-BRCA (*P* = 6.23 × 10^−14^), PLX4720-SKCM (*P* = 4.69 × 10^−11^), docetaxel-PRAD (*P* = 7.18 × 10^−3^). (c) Three bar charts of integrated gradients values for the top-contributing genes in patients a, b, and c; bars to the right indicate positive contributions to predicted Gefitinib resistance, bars to the left indicate negative contributions.

Beyond displaying the key features or genes, we further conduct the perturbation in the importance ranking of features or genes across six patient datasets. As biomarkers the rank stability of these features or genes is necessary [16]. By calculating the Spearman correlation coefficient among pairwise feature ranking (Fig. 9a). A high correlation has been identified in both feature and gene rankings. Although the top 30 features or genes (Figs S7 and S8) in resistant head show variability in rankings across different patient datasets, the influence of these features on model predictions is approximately the same, at least in the same direction of the impact (e.g. features scFoundation_1491, scFoundation_3031, scFoundation_737, *RPS18*, *RPS29*, *RPL41*).

Overall, scATD effectively identifies key features and demonstrates robust interpretability across multiple datasets, providing a higher level of assurance for the selection of critical features and genes as potential biomarkers [16], recalling the biomedical community’s concerns regarding the robustness of interpretation technic application in the biomedical field [16].

### Gene scoring and prognosis risk analysis

The identification of biomarkers is significant as they can serve as potential indicators for assessing patients’ disease survival times and as measures for evaluating the risk of adverse outcomes. RNA-seq expression profiles and clinical prognosis data from five cancer types (LUAD, PAAD, BRCA, SKCM, PRAD) in TCGA were used to identify the top 30 importance genes for each cohort. These genes were applied to Cox proportional hazards models, stratifying patients into high- and low-risk groups based on median risk levels. A clear distinction in survival outcomes between groups (*P* < 0.01) was observed across datasets, This result further validates the effectiveness of scATD in identifying genes as biomarkers related to disease risk prognosis (Fig. 9b).

Additionally, based on gene attribution values, scATD can calculate a drug resistance probability score for each patient by normalizing the sum of all gene attribution values to a range of 0–1. It also evaluates the importance and contribution direction of each gene for individual patients. Results from the TCGA LUAD cohort, including three long-term survivors (OS-0-Max3), three deceased patients with the longest survival (OS-1-Max3), and three with the shortest survival (OS-1-Min3), reveal differences in patient-specific unique gene importance rankings, gene expression levels, and their impact on Gefitinib resistance predictions (Table S9, Fig. 9c).

In the OS-0-Max3 group (Fig. S9a), all patients were survivors with near-maximum follow-up times but had varying Gefitinib resistance probabilities in the scATD-sf-dist model: Patient A (0.08), Patient B (0.37), and Patient C (0.4). *DSP*, encoding desmosomal proteins critical for cytoskeletal stability, emerged as a top-10 important gene. The downregulation of *DSP* has been linked to tumor invasion [50]. This may trigger or be associated with Gefitinib resistance. Patients A and C had lower *DSP* expression (3.69, 2.94) than Patient B (7.76). Positive *DSP* attribution values for patients A and C indicated its contribution to model predicting Gefitinib resistance, and B’s negative attribution value suggested an opposite direction. Moreover, in the OS-1-Max3 and OS-1-Min3 groups, genes (such as *MSMB*, *BPIFA1*, *FGG*, and *CPA3*) also exhibited differential expression (Fig. S9b and c). These differences can be reflected in the gene attribution values, illustrating how variations in gene expression impact resistance prediction.

This further demonstrates that the model’s interpretability aligns with biomedical knowledge, and more patient-specific gene information than analysis in patient cohorts (Fig. 7). This also represents the classic difference between global and local interpretation analyses [14].

## Discussion

In this research, the scATD framework utilizes multi-domain RNA-seq data-derived features extracted from RNA-seq LLMs and further enhances this latent representation via a pre-trained Res-VAE and a transfer framework to achieve high-accuracy predictions of drug responses at the single cell level (more precise resolution than bulk prediction model [51]). Through detailed comparative and ablation experiments, the efficacy of scATD has been validated, achieving SOTA results in broader single cell datasets. The primary intention of designing scATD remains to harness the powerful few-shot and even zero-shot transfer capabilities of LLMs to enhance drug response prediction. Given that high-quality data and labels are always scarce in the medical field, how to transfer high-quality latent representations from LLMs across domains (especially the unpaired biomedical data) to reduce the reliance on high-quality domain-specific labels will likely become a focal point in the era of LLMs for drug response prediction and broader biomedical fields application.

Besides, with the application of knowledge distillation, we demonstrate simultaneous improvements in both inference speed and prediction accuracy. As large models become more complex (e.g. scFoundation), they often require more computational resources, such as support from cloud servers. The distillation model enables local RNA-seq analysis, predicting single-cell drug resistance efficiently while protecting privacy.

Furthermore, compared to models trained on supervised single-cell drug response labels derived from individual patients, domain adaptive transfer learning exhibits relatively robust generalization capabilities, even without relying on single-cell labels. However, both scATD and the comparison domain adaptive model demonstrated suboptimal prediction accuracy on certain datasets. This suggests stricter evaluation of model generalization. Recent studies focus on accurately evaluating the true generalization ability of machine learning models on biological data [17, 52]. The heterogeneity of biological data, akin to “dark matter”, presents challenges for evaluating models in experiments with limited test data. Therefore, for future research involving single-cell-level drug sensitivity testing, it is crucial to specify dataset sources and incorporate as diverse data types as possible.

Moreover, in the field of drug response prediction and more broadly in biomedical AI modeling, the practice of integrating multi-modal data to enhance feature representation has improved model prediction accuracy and task-solving capabilities [44, 51, 53, 54]. Although scATD is a single-modality framework, the key transfer learning technique also holds the potential for aligning and integrating multi-modal data [55], which is regarded as a cornerstone for building multimodal models. Additionally, compared to AI modeling approaches that incorporate multiple modalities, single-modality scATD has advantages in clinical deployment. After all, acquiring multi-omics data through sequencing and medical imaging may increase clinical costs and complicate model interpretability. However, given the importance of multi-omics data in elucidating clinicopathological information [56], designing effective feature integration models while reducing the reliance of models on extensive multi-omics data collection is a direction worthy of further investigation.

It is noteworthy that, feature attribution algorithms like IG lack causal properties and their explanations may not align with existing clinical trial outcomes. After all, hidden features learned by the model are difficult to interpret as actual complex pathological mechanisms. In our experiment, some gene expressions exhibit a near-linear relationship with feature attribution values, which oversimplifies real-world scenarios. Additionally, scATD’s interpretable architecture is not fully symmetric.

These phenomena may stem from the feature attribution algorithm or inherent model biases [57]. Notably, scATD, as a single-modality framework trained solely on RNA-seq data, cannot directly capture the complexities of proteomics (e.g. subcellular localization and protein–protein interactions), host–pathogen interactions, pharmacology, or small molecule–cancer associations, which have been shown in previous studies to be closely related to cancer initiation, progression, and prognosis [58–61]. Thus, IG-based attributions should be viewed as indicative of potential correlations rather than definitive mechanistic markers. The inferred biomarkers remain hypothetical and require further biological and clinical validation to confirm their relevance and efficacy.

Despite these limitations, scATD explores gene-drug resistance patterns at the local level, demonstrates zero-baseline issues, and provides a framework for more scientific evaluation of the selected biomarkers. Hence, future work may explore advanced feature attribution methods and causal inference frameworks [62] to improve interpretation robustness and better align with gene regulation and biological processes.

## Conclusions

In summary, scATD achieves SOTA results in single-cell drug response prediction, outperforming multiple advanced models in more independent datasets. Experiments assessing inference speed show that scATD achieves faster inference than other baseline models. Experiments also reveal challenges for scATD and other RNA-seq-based models in transferring from cell line data to certain single-cell datasets, with some AUCs falling below 0.5. Direct training on single-cell data also shows poor generalization. Ablation studies confirm the effectiveness of the VAE and AdaIN modules. Further tests across different predictive tasks show that AdaIN-based transfer learning struggles when faced with heterogeneous labels. These results provide a more comprehensive evaluation of both transfer and non-transfer algorithms, as well as the effectiveness of RNA-seq-based large models for single-cell drug sensitivity prediction.

scATD further integrates feature attribution techniques to identify biomarkers (key features and genes) with robust importance rankings and their relationships to drug responses. These interpretable qualities make scATD appropriate for analyzing potential gene-driven drug responses and understanding the decision-making mechanisms of the deep learning model.

## Code and data availability

The bulk of cancer drug response data are downloaded from https://github.com/OSU-BMBL/scDEAL. All scRNA-seq datasets utilized in this study are available in the GEO database (https://www.ncbi.nlm.nih.gov/geo/) under the following accession codes: GSE108383, GSE108397, GSE111014, GSE112274, GSE117872, GSE131984, GSE140440, GSE149214, GSE149383, GSE163836, GSE169246, GSE186960, GSE202234, GSE223779, and GSE137829.

Panglao scRNA-seq data used for Res-VAE model pretraining are downloaded from https://github.com/TencentAILabHealthcare/scBERT. Additionally, the expression matrices and clinical information for five cancer types (LUAD, PAAD, BRCA, SKCM, and PRAD) were downloaded from the TCGA database (https://portal.gdc.cancer.gov/). Details of the downloading and processing procedures for the BRCA_RECIST and PAAD_RECIST datasets are provided in the Supplementary Materials. Besides, the raw data and corresponding LLM feature embeddings are available on figshare at https://figshare.com/articles/software/scATD/27908847. The Code in this experiment is located at https://github.com/doriszmr/scATD.

## Glossary and list of abbreviations

**Table:** 

**Abbreviation**	**Common terms**
scRNA	Single-cell RNA sequencing
LLMs	Large Language Models
Res-VAE	Residual Variational Autoencoder
Bi-AdaIN	Bidirectional Adaptive Instance Normalization
SOTA	state-of-the-art
TCGA	The Cancer Genome Atlas
LUAD	lung adenocarcinoma
PAAD	pancreatic adenocarcinoma
BRCA	breast invasive carcinoma
SKCM	skin cutaneous melanoma
PRAD	prostate adenocarcinoma
BERT	Bidirectional Encoder Representations from Transformers
ReLU	Rectified Linear Unit
REC	Reconstruction
KLD	Kullback–Leibler divergence
COD	cosine similarity-based distillation
MLP	Multilayer Perceptron
MMD	Maximum Mean Discrepancy
SMOTE	Synthetic Minority Over-sampling Technique
MCC	Matthews correlation coefficient
TPE	Tree-structured Parzen Estimator
AUROC	The areas under the ROC
AUPRC	Area Under the PRC
UMAP	Uniform Manifold Approximation and Projection
IG	Integrated Gradients
ICL	In-Context Learning
SFT	supervised fine-tuning
GO	Gene Ontology
ADDA	Adversarial Discriminative Domain Adaptation

**Key Points**

- Developed a transfer learning framework with pre-trained LLMs for label-free single-cell drug response prediction.
- Reduced model size via knowledge distillation, significantly improving efficiency.
- Used stringent background reference samples as baselines in feature attribution to identify key genes and drug resistance mechanisms.

## Supplementary Material

Supplementary_material_bbaf268
