# Supplementary material for: scATD: a high-throughput and interpretable framework for single-cell cancer drug resistance prediction and biomarker identification
Source: Brief Bioinform. 2025 Jun 12;26(3):bbaf268. doi: 10.1093/bib/bbaf268 (PMC12159290; doi:10.1093/bib/bbaf268)
Supplement: Supplementary_material_bbaf268 [file supplementary_material_bbaf268.docx]

**SUPPLEMENTARY MATERIAL**

**Supplementary Methods**

1. **Workflow for Data Preprocessing and Feature Extraction from scFoundation**

Step 1: Preprocessing RNA-seq Gene Expression Data
Download the RNA-seq gene expression dataset in h5ad format containing unnormalized raw counts. Process the data into a matrix with rows representing cells and columns representing genes (using Gene Symbol). Using the scFoundation gene vocabulary (refer to <https://github.com/doriszmr/scATD>), perform the following steps:

1. Filter genes: Remove genes that are not included in the scFoundation gene vocabulary.
2. Impute missing genes: Add genes that are present in the scFoundation vocabulary but missing in the RNA-seq dataset, with expression values filled as zero.
3. Rearrange columns: Reorder the gene columns in the RNA-seq dataset to match the sequence in the scFoundation gene vocabulary.

Step 2: Feature Inference
Perform feature inference on the RNA-seq dataset processed in Step 1. Following the recommendations outlined in the scFoundation publication, use different precision parameters for inference depending on the type of dataset:

- For bulk RNA-seq data, apply parameters tailored to bulk resolution.
- For single-cell RNA-seq data, apply parameters tailored to single-cell resolution.

Detailed parameter configurations and recommendations are available at <https://github.com/doriszmr/scATD>.

The final output is a feature matrix, $X\in\mathbb{R}^{N\times F}$, where $N$ represents the number of cell lines in bulk data or the number of cells in single-cell data, $F$ represents the feature embedding dimension, which is equal to 3072.

- 1. **Workflow for Data Preprocessing and Feature Extraction from geneformer**

Step 1: Preprocessing RNA-seq Gene Expression Data
Download the RNA-seq gene expression dataset in h5ad format containing unnormalized raw counts. Process the data into a matrix with rows representing cells and columns representing genes (using Ensembl ID). Using the geneformer gene vocabulary (refer to <https://github.com/doriszmr/scATD>), perform the following steps:

1. Filter genes: Remove genes that are not included in the Geneformer gene vocabulary.
2. Select top expressed genes: For each cell, retain the top 2048 genes with the highest expression levels. If the number of retained genes is fewer than 2048, fill the remaining positions with padding tokens.
3. Rearrange columns: Ensure that the retained genes are indexed in the same order as in the Geneformer gene vocabulary.

Step 2: Feature Inference
Perform feature inference on the RNA-seq dataset processed in Step 1. Following the recommendations outlined in the geneformer publication, use the same precision parameters for both bulk or single-cell RNA-seq data. Detailed parameter configurations and recommendations are available at <https://github.com/doriszmr/scATD>.

The final output is a feature matrix, $X\in\mathbb{R}^{N\times F}$, where $N$ represents the number of cell lines in bulk data or the number of cells in single-cell data, $F$ represents the feature embedding dimension, which equal to 256 (gf-6L-30M-original) or 512 (gf-12L-30M-i2048).

- 1. **Workflow for Data Preprocessing and Experiment Detail in scDEAL**

Step 1: Preprocessing RNA-seq Gene Expression Data
Download the RNA-seq gene expression dataset in h5ad format containing unnormalized raw counts. Process the data into a matrix with rows representing cells and columns representing genes (not specified, using Gene Symbol or Ensembl ID). Perform the following steps:

1. Remove cells with fewer than 200 genes expressed and genes expressed in fewer than 3 cells. Exclude cells where mitochondrial gene content exceeds 5%.
2. Rearrange columns: Keep cells with the number of expressed genes between 0 and 20000.
3. Normalize total counts per cell, Adjust the total expression of each cell to a unified value (10,000) to correct for sequencing depth variations.
4. For each expression value conducting log-transformation ($log(1+x)$).
5. If performing inference using a previously trained scDEAL model, ensure that the gene names and their order in the inference dataset match those in the training dataset.

The above data preprocessing methods follow the default settings for general data preprocessing in scDEAL. For detailed parameter configurations and code, please refer to the scDEAL official online documentation: https://github.com/OSU-BMBL/scDEAL.

Step 2: model training parameter setting

For bulk model training phase parameter training, we set the default parameter present in scDEAL official online documentation: <https://github.com/OSU-BMBL/scDEAL>. For the single cell domain transfer learning phase, we set parameter *dimreduce* (Encoder model type) as DAE (Denoise Auto Encoder), parameter *mod* as new (Embed the cell type label to regularize the training), other parameters are set as default present in scDEAL official online documentation: <https://github.com/OSU-BMBL/scDEAL>.

- 1. **Workflow for Data Preprocessing and Experiment Detail in SCAD**

We use features extracted from scFoundation as SCAD input and conduct experiments on single-cell datasets, which provides advantages over directly using RNA-seq gene expression data as input, as leading research (PMID: 38844628) demonstrate.

Step 1: Preprocessing RNA-seq Gene Expression Data and Extracting Feature from scFoundation
Following the ‘Step 1: Preprocessing RNA-seq Gene Expression Data’ and ‘Step 2: Feature Inference
’ in scFoundation section, extracting feature from scFoundation.
Step 2: Conducting experiments within the SCAD transfer learning framework

Following the steps detailed in the scFoundation online instructions for SCAD model training (https://github.com/biomap-research/scFoundation/tree/main/SCAD), then evaluating the model's performance across sixteen datasets.

- 1. **Workflow for Data Preprocessing and Experiment Step in DrugFormer**

Data preprocessing and model construction detail can be found in DrugFormer online instruction (<https://github.com/QSong-github/DrugFormer>). Then valuating the model's performance across sixteen datasets. Note experiments in DrugFormer is supervised learning.

- 1. **Workflow for Data Preprocessing and Experiment Detail in scATD**

For the scATD-gf and scATD-sf model, follow the above geneformer and scfoundation data preprocess and feature extraction methods to get the corresponding feature matrix. Then follow the methods described in the main text, conducting model training, inference, and key feature selection. For more details, please refer to <https://github.com/doriszmr/scATD>.

For the scATD-sf-dist model,Follow Step 1: Preprocessing RNA-seq Gene Expression Data in the scFoundation framework to perform data preprocessing. Then follow the methods described in the main text, conducting model training, inference, and key gene selection. For more details, please refer to <https://github.com/doriszmr/scATD>.

- 1. **Data Augmentation Strategy Based on VAE**

In addition to the SMOTE algorithm, the pretraining scRNA-seq VAE model was deployed to execute data augmentation. The input to the VAE model consists of a training set derived from a 5-fold partition of bulk data, where samples are selected through uniform random sampling. The reconstructed outputs from the VAE model's decoder are considered as the new synthesized dataset. Compared to the SMOTE technique, which linearly interpolates between neighboring data points for data augmentation, VAE-based augmentation can sample around the center of input data following the latent distribution learned by the VAE model. Specifically, the sampling ratio for augmenting minority and majority classes adheres to the following formula:

1. Given majority class count $M$ and minority class count $m$, Compute the Class Difference Ratio:

$R=\frac{M-m}{M}$ (1)

1. Set Initial Multipliers Based on $R$:

$\left( \alpha,\beta\right)=\left\{ \begin{aligned} \left( 1,\frac{M}{m} \right), if R<0.3(case 1) \\ \left( 1.5,\beta_{desired} \right),if R\geq0.3(case 2) \end{aligned} \right.$ (2)

where $\alpha$ is the Majority Multiplier, $\beta$ is the Minority Multiplier. If satisfied $case 1$, $\alpha$ and $\beta$ are the final ratio values. Additionally, $\beta_{\text{desired}}$ is defined as $\beta_{\text{desired}}=\frac{\alpha M}{m}$.

1. Apply Constraints to the Minority Multiplier $\beta$:

$\beta=min(\max\left( \beta_{desired},1.2 \right),4)$ (3)

1. Update Adjusted Class Counts:

$M^{'}=\alpha M, m^{'}=\beta m$ (4)

1. Check and Adjust for Imbalance (only for Case 2):

$$\text{If }m'<0.5M'\text{, then reset }\alpha=1\text{ and recompute:}$$

$\beta_{new}=\min\left( \max\left( \frac{M}{m},1.2 \right),4 \right)$ (5)

$m^{'}=\beta_{new}\times m$ (6)

1. The final values for $\alpha$ and $\beta$ are:

$\alpha_{final}=\alpha, \beta_{final}=\left\{ \begin{aligned} \beta, if no adjustment needed \\ \beta_{new}, if step5 adjustment applied \end{aligned} \right.$ (7)

The fixed coefficients in the formula are selected based on experience and experimental results. After VAE augmentation, the Tomek link is also used to clarify the boundary between the two classes. VAE-based augmentation is deployed in the scATD-gf, scATD-sf, scATD-sf-dist.

**H. BRCA_RECIST Data Downloading and Processing**

The clinical information for the BRCA_RECIST dataset was downloaded from the Genomic Data Commons (GDC) portal (<https://portal.gdc.cancer.gov/>) by querying clinical records within the TCGA-BRCA project. Specifically, clinical data were identified under the "Clinical" category, which contained information on 1,098 patients and included a total of 2,288 files.

Patient drug responses initially recorded according to RECIST criteria were categorized: "Complete Response" or "Partial Response" was designated as "Response"; "Stable Disease" or "Progressive Disease" was designated as "Non Response."

Additionally, RNA-seq data for the BRCA_RECIST dataset were downloaded from the GDC portal.

**I. PAAD_RECIST Data Downloading and Processing**

RNA-seq data and drug response labels for the PAAD_RECIST dataset were directly obtained from the publicly available dataset shared by Adeolu Ogunleye et al. on GitHub (<https://github.com/chayanitpiy/gemcitabine_PAAD>). This dataset includes RNA-seq data from 65 patients along with corresponding gemcitabine response labels.

**J. Implementation of Bi-AdaIN and MMD in scATD**

In the scATD framework, (Bidirectional Adaptive Instance Normalization (Bi-AdaIN) and Maximum Mean Discrepancy (MMD) methods are separately utilized for distinct sub-models to achieve transfer learning from bulk RNA-seq data to single-cell RNA-seq data.

Bidirectional Adaptive Instance Normalization (Bi-AdaIN) Method:

- Training Phase: During training, the scATD-sf and scATD-gf sub-models apply the Bi-AdaIN method for transfer learning. Specifically, 10 independent models are separately trained, each exclusively on bulk RNA-seq data with their corresponding drug response labels, covering 10 distinct drug-disease combinations. During this stage, the transfer learning dataset utilized is derived from the Panglao single-cell dataset, previously employed during the VAE pre-training phase.

- Inference Phase: At inference time, for each of the different GSE single-cell RNA-seq datasets, the method requires only the calculation of feature (extraction from LLM) means and standard deviations for the specific drug-disease model. These statistical parameters are used solely for the statistical alignment of single-cell RNA-seq features required for model inference, without utilizing the labels from these datasets.

Maximum Mean Discrepancy (MMD) Method:

- Training Phase: The MMD method is predominantly utilized in the scATD-dist-sf sub-model. It achieves domain adaptation by minimizing the distributional differences between the source domain (bulk RNA-seq data with labels) and the target domain (single-cell RNA-seq data without labels). Importantly, the single-cell RNA-seq data used for adaptation do not provide drug-response labels, which remain unseen throughout training.

- Inference Phase: At inference time, the adapted model can predicts drug responses on single-cell RNA-seq datasets. However, in principle, for a new, unseen patient, the domain adaptation process should be repeated using the patient’s single-cell RNA-seq data (without labels) to ensure accurate alignment and prediction.

**Supplementary Results**

**Table S1. Benchmark protocol in Drug reaction prediction**

| **Protcol name** | **Model framework** | **Training data with label** | **Domain prediction data** | **Domain-adaptive transfer learning^a^** |
| --- | --- | --- | --- | --- |
| scDEAL | Conditional DAE | Bulk level | Single cell level | Yes |
| SCAD | DANN | Bulk level | Single cell level | Yes |
| TMO-Net | VAE | Bulk level | Bulk level | No |
| ScFoundation-SCAD | LLM-DANN ^b^ | Bulk level | Single cell level | Yes |
| DrugFormer | Graph Transformer | Single cell level | Single cell level | No |

^a^: The drug sensitivity prediction model employs explicit transfer learning architectures, such as MMD or DANN, only if it involves explicit domain alignment. In comparison, A model that is simply pre-trained in one domain and then directly applied in another without domain alignment does not constitute explicit use of a transfer learning architecture.

^b^: RNA-Seq Large Language Model (ScFoundation) pretraining & GAN domain transfer (SCAD).

**Table S2. AUC Results for Samples Filtered by AUC > 0.5**

| **Metric** | **scDEAL**  **(11)** | **SCAD**  **(9)** | **scATD-gf**  **(9)** | **scATD-sf**  **(14)** | **scATD-sf-dist**  **(16)** |
| --- | --- | --- | --- | --- | --- |
| Mean | 0.68 | 0.64 | 0.60 | 0.66 | **0.71** |
| Median | 0.67 | 0.64 | 0.57 | 0.66 | **0.68** |
| Standard Deviation | 0.1 | 0.12 | **0.08** | 0.1 | 0.16 |

**Table S3. Mean and standard deviation of the five-fold cross-validation results for model inference**

| **Dataset** | **Model** | **Metric** | **MCC** | **AUROC** | **F1_Score** | **Precision** | **Recall** |
| --- | --- | --- | --- | --- | --- | --- | --- |
| GSE112274 | scDEAL | Mean | 0.02 | 0.75 | 0.02 | 0.8 | 0.01 |
|  |  | Std | 0.01 | 0.05 | 0.01 | 0.45 | 0.01 |
|  | scATD-gf | Mean | 0 | 0.68 | **0.96** | 0.93 | 1 |
|  |  | Std | 0 | 0.08 | 0 | 0 | 0 |
|  | scATD-sf | Mean | 0.11 | 0.67 | 0.77 | 0.95 | **0.64** |
|  |  | Std | 0.06 | 0.07 | 0.03 | 0.01 | 0.04 |
|  | scATD-sf-dist | Mean | **0.31** | **0.94** | 0.77 | **0.99** | 0.63 |
|  |  | Std | 0.07 | 0.05 | 0.04 | 0.01 | 0.05 |
| GSE140440 | scDEAL | Mean | **0.5** | 0.79 | **0.74** | 0.77 | 0.72 |
|  |  | Std | 0.14 | 0.07 | 0.06 | 0.09 | 0.05 |
|  | scATD-gf | Mean | 0.08 | 0.57 | 0.67 | 0.51 | 0.96 |
|  |  | Std | 0.12 | 0.08 | 0.02 | 0.02 | 0.04 |
|  | scATD-sf | Mean | 0.16 | 0.71 | 0.68 | 0.52 | **0.99** |
|  |  | Std | 0.05 | 0.03 | 0 | 0.01 | 0.02 |
|  | scATD-sf-dist | Mean | 0.496 | **0.82** | 0.65 | **0.88** | 0.52 |
|  |  | Std | 0.1 | 0.06 | 0.1 | 0.06 | 0.12 |
| GSE223779 | scDEAL | Mean | 0.2 | 0.6 | 0.58 | 0.63 | 0.54 |
|  |  | Std | 0.02 | 0 | 0.01 | 0.01 | 0.01 |
|  | scATD-gf | Mean | 0 | 0.75 | 0.68 | 0.52 | 1 |
|  |  | Std | 0 | 0 | 0 | 0 | 0 |
|  | scATD-sf | Mean | 0.42 | 0.77 | 0.71 | 0.73 | 0.7 |
|  |  | Std | 0.01 | 0.01 | 0.01 | 0.01 | 0.01 |
|  | scATD-sf-dist | Mean | **0.51** | **0.85** | **0.73** | **0.83** | **0.65** |
|  |  | Std | 0 | 0 | 0 | 0 | 0.01 |

**Table S4. Evaluation results of DrugFormer**

| **No.** | **Dataset** | **AUROC** | **AUPRC** | **F1 score** |
| --- | --- | --- | --- | --- |
| 1 | GSE117872_HN120 | **0.9963** | 0.954 | 0.9682 |
| 2 | GSE117872_HN137 | 0.7008 | 0.7184 | 0.8323 |
| 3 | GSE112274 | 0.798 | 0.0729 | 0.076 |
| 4 | GSE140440 | 0.8249 | 0.515 | 0.4677 |
| 5 | GSE149383 | 0.7631 | 0.5863 | 0.5718 |
| 6 | GSE108383_X451 | 0.7067 | 0.4394 | 0.136 |
| 7 | GSE108383_A375 | 0.6285 | 0.6 | 0.7435 |
| 8 | GSE163836 | **0.9999** | 0.9887 | **0.9932** |
| 9 | GSE186960 | **0.9974** | 0.8183 | 0.8849 |
| 10 | GSE223779 | **0.9995** | 0.9865 | **0.9912** |
| 11 | GSE108397 | **0.9984** | 0.9726 | 0.9832 |
| 12 | GSE149214 | 0.7428 | 0.5933 | 0.6482 |
| 13 | GSE202234 | 0.749 | 0.605 | 0.4998 |
| 14 | GSE131984 | 0.6175 | 0.7544 | 0.6075 |
| 15 | GSE111014 | **0.9926** | 0.9408 | 0.9578 |
| 16 | GSE169246 | 0.5007 | 0.4502 | 0 |

**Table S5. Results of ablation experiment**

| **Dataset** | **scATD-gf** | | **scATD-sf** | | **Geneformer (Non_VAE)** | | **ScFoundation (Non_VAE)** | | **scATD-gf (Non_adain)** | | **scATD-sf (Non_adain)** | |
| --- | --- | --- | --- | --- | --- | --- | --- | --- | --- | --- | --- | --- |
|  | **AUC** | **PR** | **AUC** | **PR** | **AUC** | **PR** | **AUC** | **PR** | **AUC** | **PR** | **AUC** | **PR** |
| GSE117872_HN120 | **0.49** | 0.03 | **0.56** | 0.37 | 0.44 | 0.3 | **0.56** | **0.4** | 0.46 | **0.32** | 0.21 | 0.22 |
| GSE117872_HN137 | **0.57** | **0.37** | **0.58** | **0.34** | 0.46 | 0.26 | 0.53 | 0.29 | 0.46 | 0.28 | 0.43 | 0.25 |
| GSE112274 | **0.68** | **0.97** | **0.66** | **0.96** | 0.27 | 0.87 | 0.48 | 0.92 | 0.35 | 0.89 | 0.17 | 0.84 |
| GSE140440 | **0.56** | **0.56** | 0.71 | 0.65 | 0.55 | 0.52 | **0.77** | **0.75** | 0.51 | 0.51 | 0.72 | 0.69 |
| GSE149383 | 0.51 | 0.57 | **0.55** | **0.61** | 0.49 | 0.53 | 0.48 | 0.56 | **0.56** | **0.61** | 0.35 | 0.49 |
| GSE108383_X451 | 0.42 | 0.56 | **0.66** | **0.7** | 0.36 | 0.49 | 0.5 | 0.57 | **0.52** | **0.58** | 0.31 | 0.45 |
| GSE108383_A375 | 0.15 | 0.26 | **0.81** | **0.74** | 0.09 | 0.4 | 0.5 | 0.4 | **0.76** | **0.54** | 0.65 | 0.61 |
| GSE163836 | 0.49 | 0.71 | **0.88** | **0.95** | **0.63** | **0.81** | 0.71 | 0.87 | 0.31 | 0.63 | 0.35 | 0.69 |
| GSE186960 | **0.67** | **0.93** | **0.52** | **0.89** | 0.54 | 0.89 | 0.15 | 0.76 | 0.29 | 0.8 | 0.23 | 0.78 |
| GSE223779 | **0.75** | **0.73** | 0.77 | 0.73 | 0.64 | 0.63 | **0.81** | **0.83** | 0.6 | 0.6 | 0.67 | 0.66 |
| GSE108397 | **0.5** | **0.49** | **0.62** | **0.61** | 0.36 | 0.4 | 0.5 | 0.5 | 0.39 | 0.42 | 0.11 | 0.32 |
| GSE149214 | 0.52 | 0.56 | **0.55** | **0.59** | 0.51 | 0.52 | 0.47 | 0.54 | **0.57** | **0.6** | 0.34 | 0.47 |
| GSE202234 | **0.64** | **0.64** | 0.44 | 0.43 | 0.41 | 0.43 | **0.46** | **0.46** | 0.54 | 0.55 | 0.38 | 0.4 |
| GSE131984 | 0.45 | 0.36 | **0.68** | **0.59** | **0.47** | **0.43** | 0.5 | 0.39 | 0.29 | 0.29 | 0.21 | 0.26 |
| GSE111014 | **0.42** | **0.45** | **0.66** | **0.61** | 0.4 | 0.44 | 0.58 | 0.55 | 0.33 | 0.4 | 0.55 | 0.54 |
| GSE169246 | **0.46** | **0.52** | 0.45 | 0.52 | 0.41 | 0.48 | 0.46 | 0.53 | 0.45 | 0.51 | **0.5** | **0.56** |
| Mean | **0.52** | **0.54** | **0.63** | **0.64** | 0.44 | 0.53 | 0.53 | 0.58 | 0.46 | 0.53 | 0.39 | 0.51 |
| Middle | **0.51** | **0.56** | **0.64** | **0.61** | 0.45 | 0.49 | 0.5 | 0.55 | 0.46 | 0.54 | 0.35 | 0.52 |

**AUC is the abbreviation for AUROC, and PR is the abbreviation for AUPRC.**

**Table S6. Computational Environment Setup**

| **Device** | **Operating System** | **Specifications** |
| --- | --- | --- |
| CPU | Ubuntu 23.04 | Intel(R) Xeon(R) Platinum 8358 CPU @ 2.60GHz |
| GPU | CentOS Linux release 7.9.2009 (Core) | NVIDIA A100 (80G) |

**Table S7. Feature KL divergence (Pre vs. Post Bi-AdaIN transfer)**

| **Dataset** | **KL_Pre_Mean** | **KL_Pre_Std** | **KL_Post_Mean** | **KL_Post_Std** |
| --- | --- | --- | --- | --- |
| GSE112274 | 10.95 | 7.66 | 8.32 | 5.26 |
| TCGA_BRCA | 14.26 | 10.57 | 5.06 | 6.12 |
| TCGA_PAAD | 14.12 | 10.12 | 5.84 | 7.84 |

**Table S8. Top 3 features and genes in the six patients from GSE137829**

| **Patient ID** | **Top3 features**  **（scATD-sf）** | **Top3 genes**  **（scATD-sf-dist）** |
| --- | --- | --- |
| Patient 1 | scFoundation_90, scFoundation_207, scFoundation_2511 | *RPS6, RPLP1, RPL41* |
| Patient 2 | scFoundation_3031, scFoundation_467, scFoundation_737 | *RPS19, RPS29, HSPB1* |
| Patient 3 | scFoundation_2925, scFoundation_90, scFoundation_916 | *RPL41, RPLP1, RPL10* |
| Patient 4 | scFoundation_90, scFoundation_737, scFoundation_1430 | *RPL41, KLK3, RPLP1* |
| Patient 5 | scFoundation_1905, scFoundation_90, scFoundation_2475 | *C6orf15, RPLP1, RPL41* |
| Patient 6 | scFoundation_2044, scFoundation_90, scFoundation_1491 | *KLK3, RPL41, RPL10* |

**Table S9. Patient sample information**

|  | **PATIENT.ID** | **PATIENT** | **OS.Time** | **OS** |
| --- | --- | --- | --- | --- |
|  | A | TCGA-78-7163 | 7248 | 0 |
| OS-0-Max3 | B | TCGA-78-8640 | 7062 | 0 |
|  | C | TCGA-49-AARQ | 6732 | 0 |
|  | D | TCGA-78-7143 | 4961 | 1 |
| OS-1-Max3 | E | TCGA-78-8662 | 3361 | 1 |
|  | F | TCGA-78-7162 | 3169 | 1 |
|  | G | TCGA-86-8672 | 19 | 1 |
| OS-1-Min3 | H | TCGA-97-7938 | 18 | 1 |
|  | I | TCGA-NJ-A4YI | 4 | 1 |


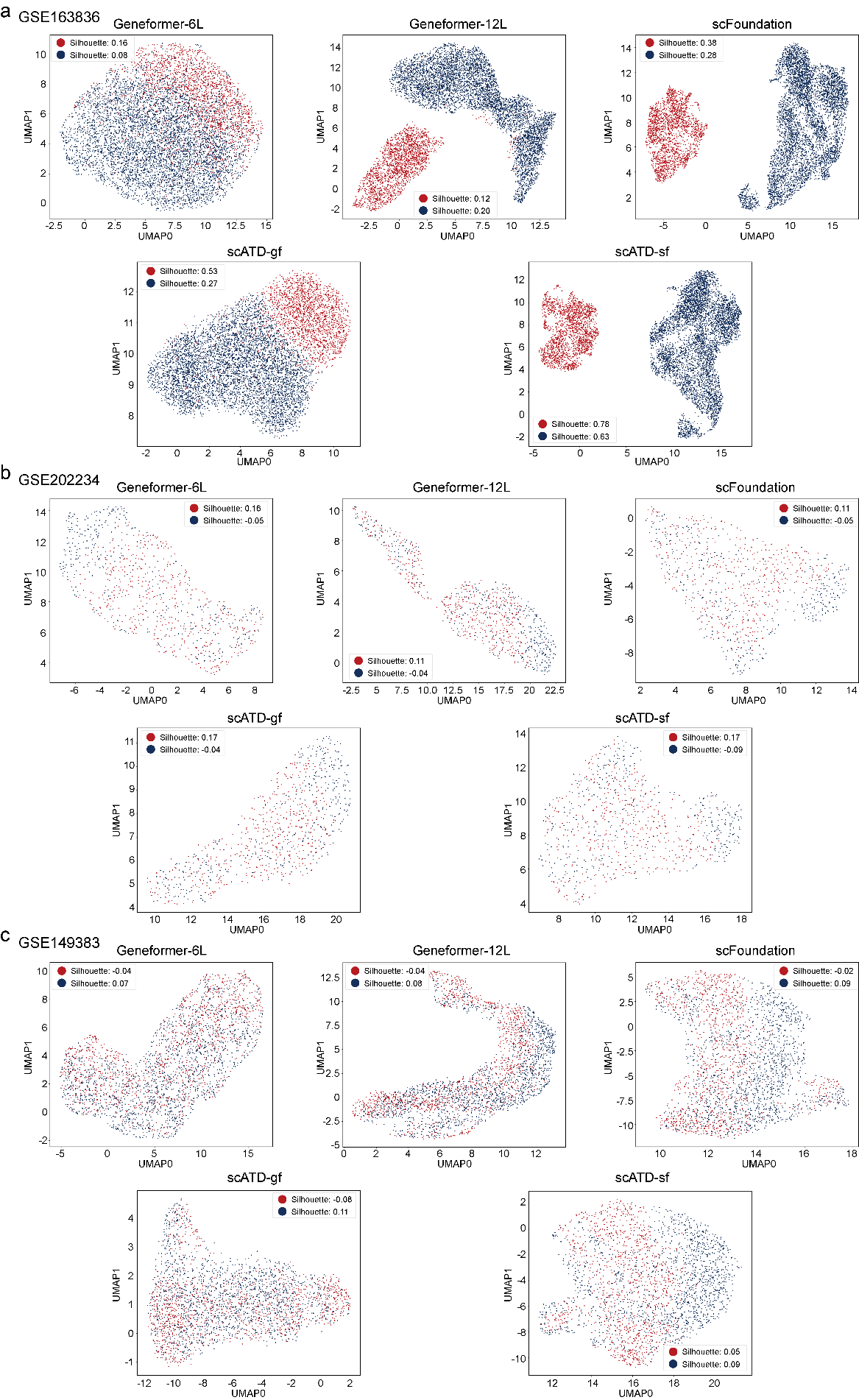


**Figure S1**. Feature latent representation visualization plot. (a). Umap-2d plot of GSE163836 (b). Umap-2d plot of GSE202234. (c). Umap-2d plot of GSE149383.

**
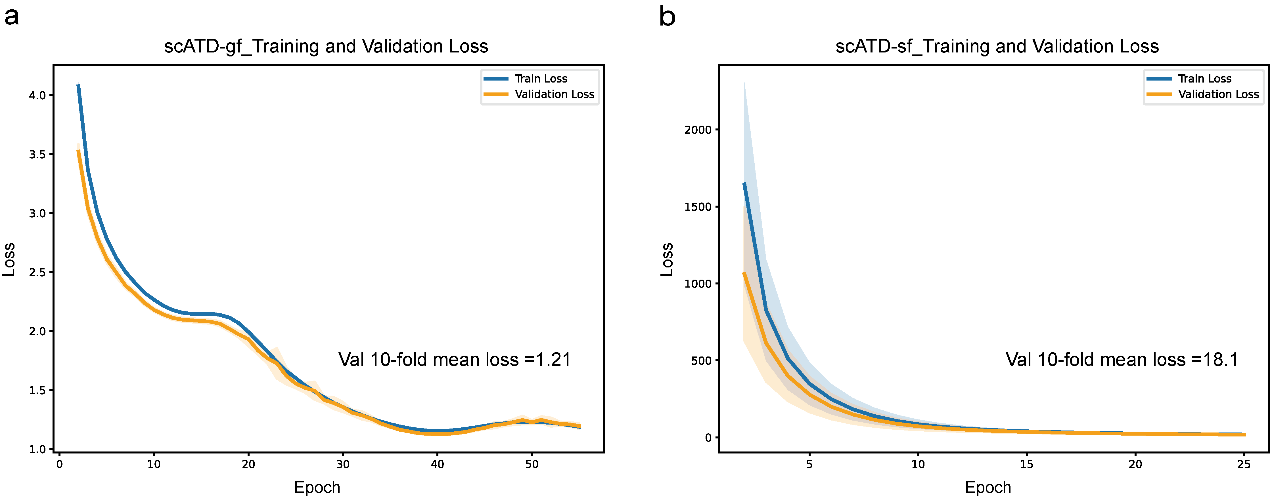
Figure S2**. The loss fitting plot of Res-VAE pretraining.


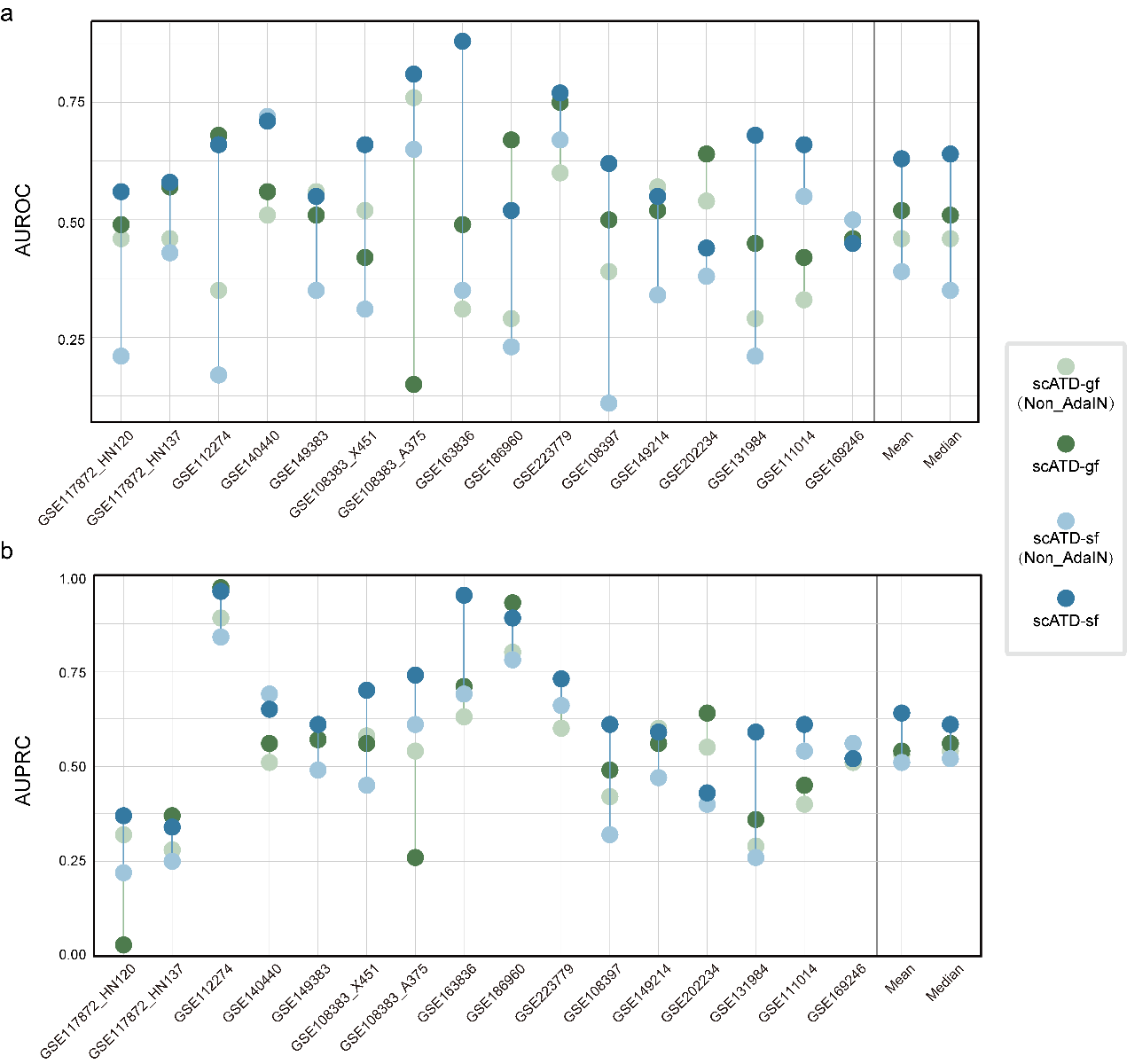


**Figure S3**. Results of ablation experiment (Bi-AdaIN vs Non_AdaIN). **(a).** AUROC results of ablation experiments across 16 single-cell datasets. **(b).** PRROC results of ablation experiments across 16 single-cell datasets.

**
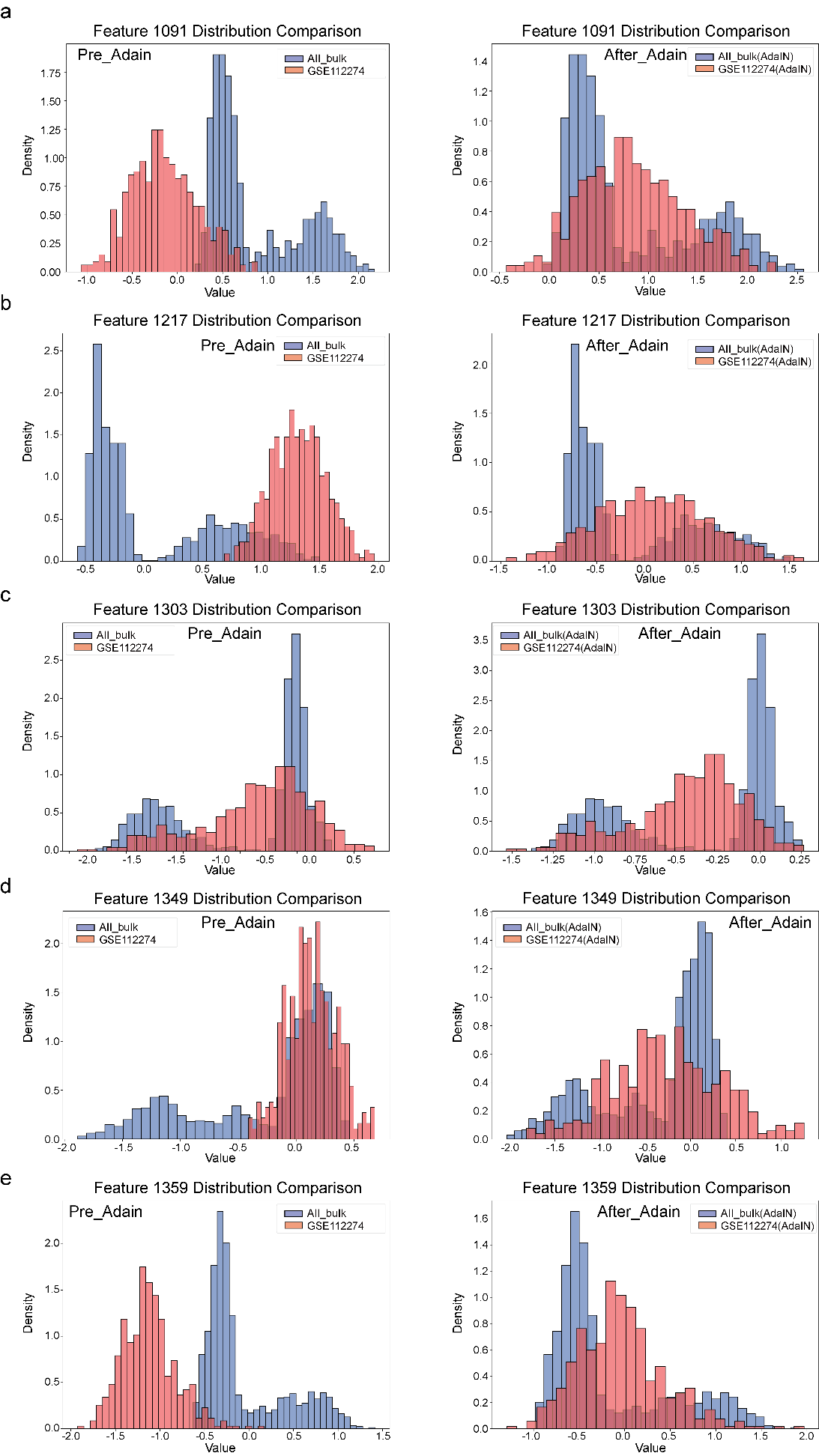
**

**Figure S4.** Numerical distributions of five features with lowest post-transfer KL divergence (Pre vs. Post Bi-AdaIN transfer)


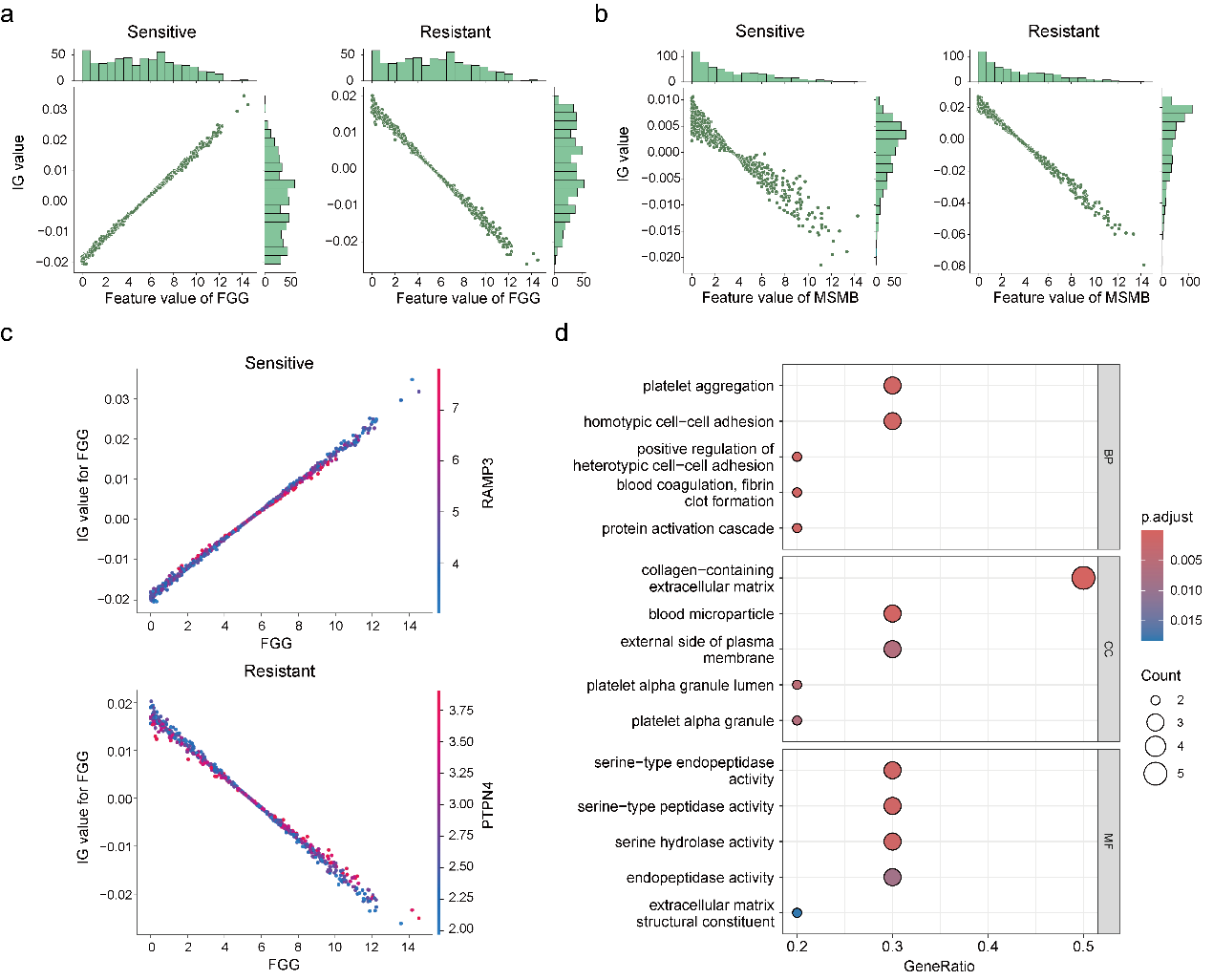


**Figure S5**. **(a).** Histogram plot of *FGG* attribution value in model two prediction head. **(b).** Histogram plot of *MSMB* attribution value in model two prediction head. **(c).** Dependence plot of *FGG*. **(d).** GO enrichment (Over-Representation Analysis) of top 10 key genes in TCGA_LUAD.



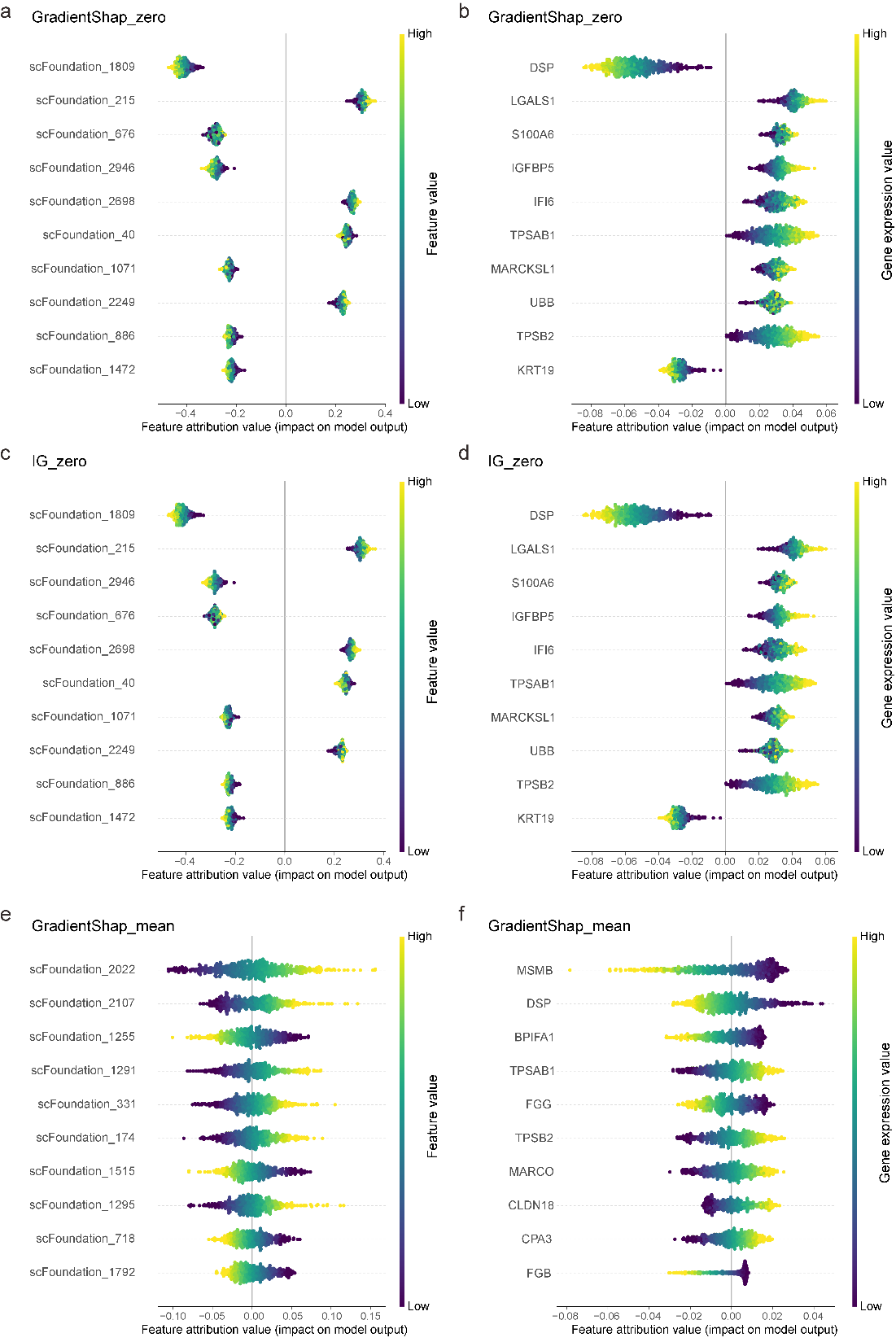


**Figure S6.** Swarm plot of top 10 feature or gene attribution value in TCGA_LUAD. **(a), (c), and (e)** are the Swarm plot of feature attribution value in scATD-sf. **(a).** Feature attribution calculation methods is GradientShap, and the baseline is zero vector. **(c).** Feature attribution calculation methods is IG, and the baseline is zero vector. **(e).** Feature attribution calculation methods is GradientShap, and the baseline is the mean of all sample’s feature as baseline. **(b), (d), and (f)** are the Swarm plot of gene attribution value in scATD-sf-dist. **(b).** gene attribution calculation methods is GradientShap, and the baseline is zero vector. **(d).** gene attribution calculation methods is IG, and the baseline is zero vector. **(f).** gene attribution calculation methods is GradientShap, and the baseline is the mean of all sample’s gene expression as baseline.


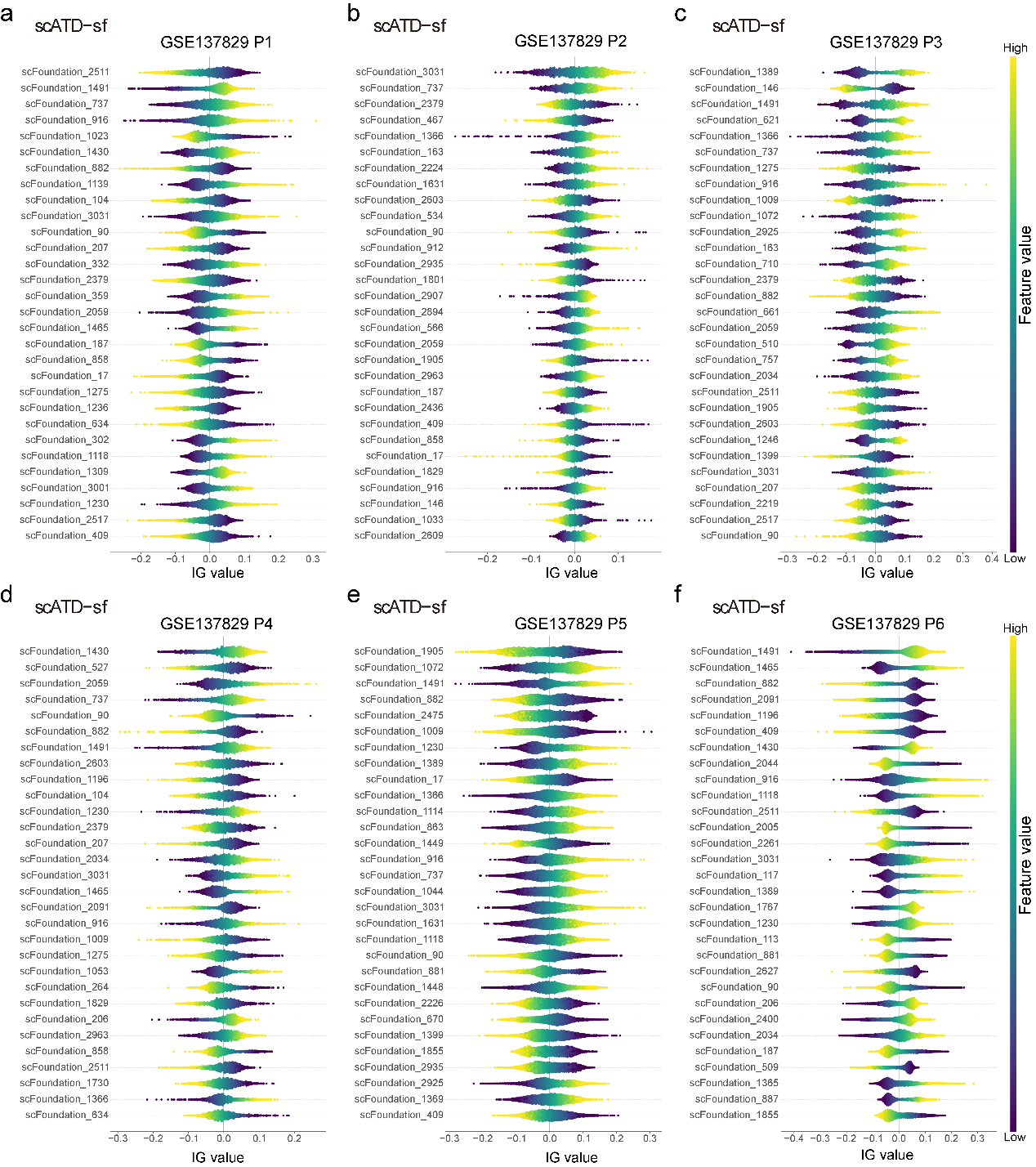


**Figure S****7**. Swarm Plot of top 30 feature Attribution in the scATD-sf Model. Features are sorted by importance from high to low. The color of the dots represents the high or low value of the feature. Besides, the x-axis represents the feature attribution value (calculated by IG), indicating whether the feature has a positive contribution (positive value) or a negative contribution (negative value) in model prediction. (a), (b), (c), (d), (e), and (f) corresponding to Patient 1-6 in GSE137829.


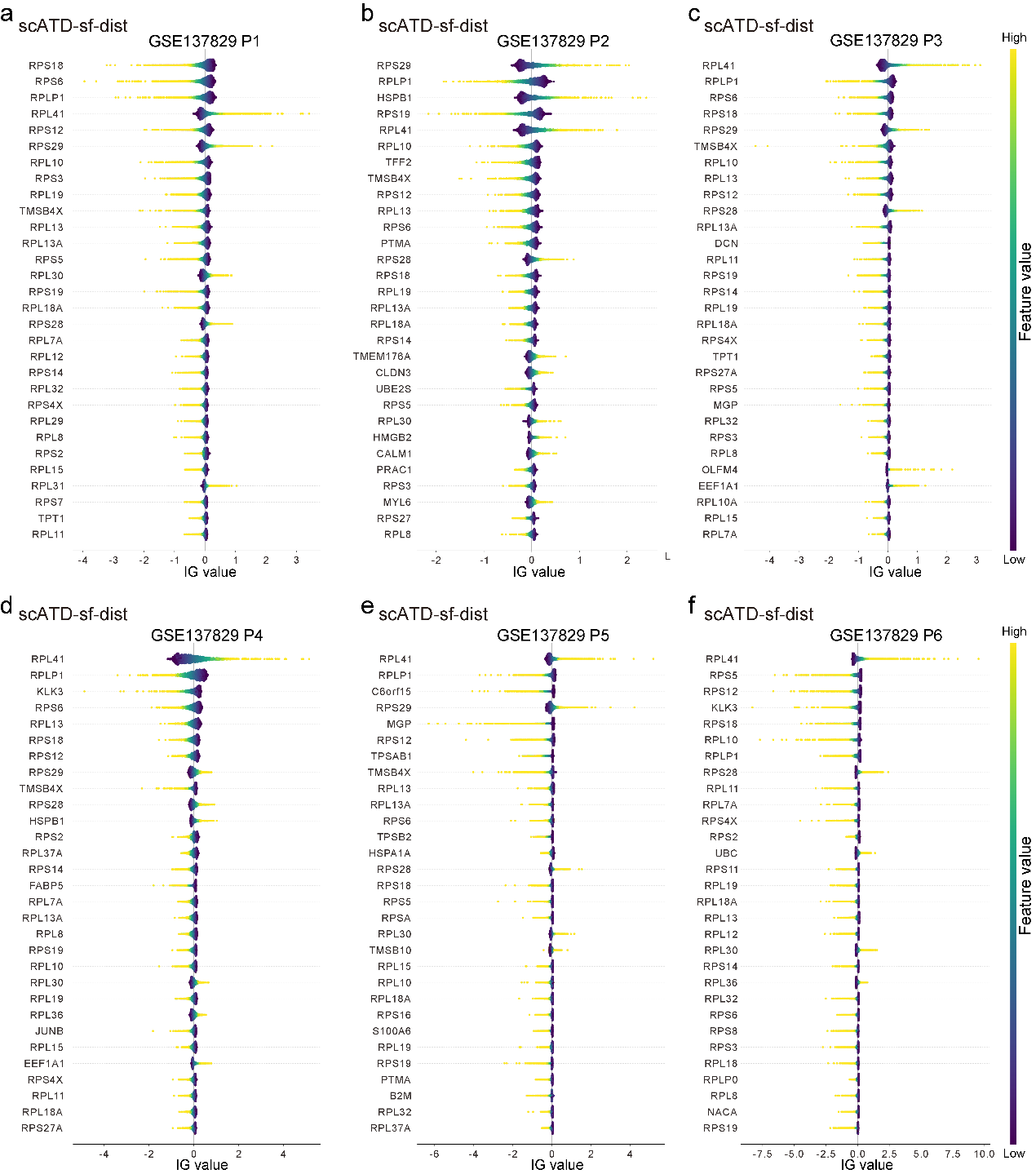


**Figure S8**. Swarm Plot of top 30 gene Attribution in the scATD-sf-dist. Genes are sorted by importance from high to low. The color of the dots represents the high or low expression value of the gene. Besides, the x-axis represents the gene attribution value (calculated by IG), indicating whether the gene has a positive contribution (positive value) or a negative contribution (negative value) in model prediction. (a), (b), (c), (d), (e), and (f) corresponding to Patient 1-6 in GSE137829.

**
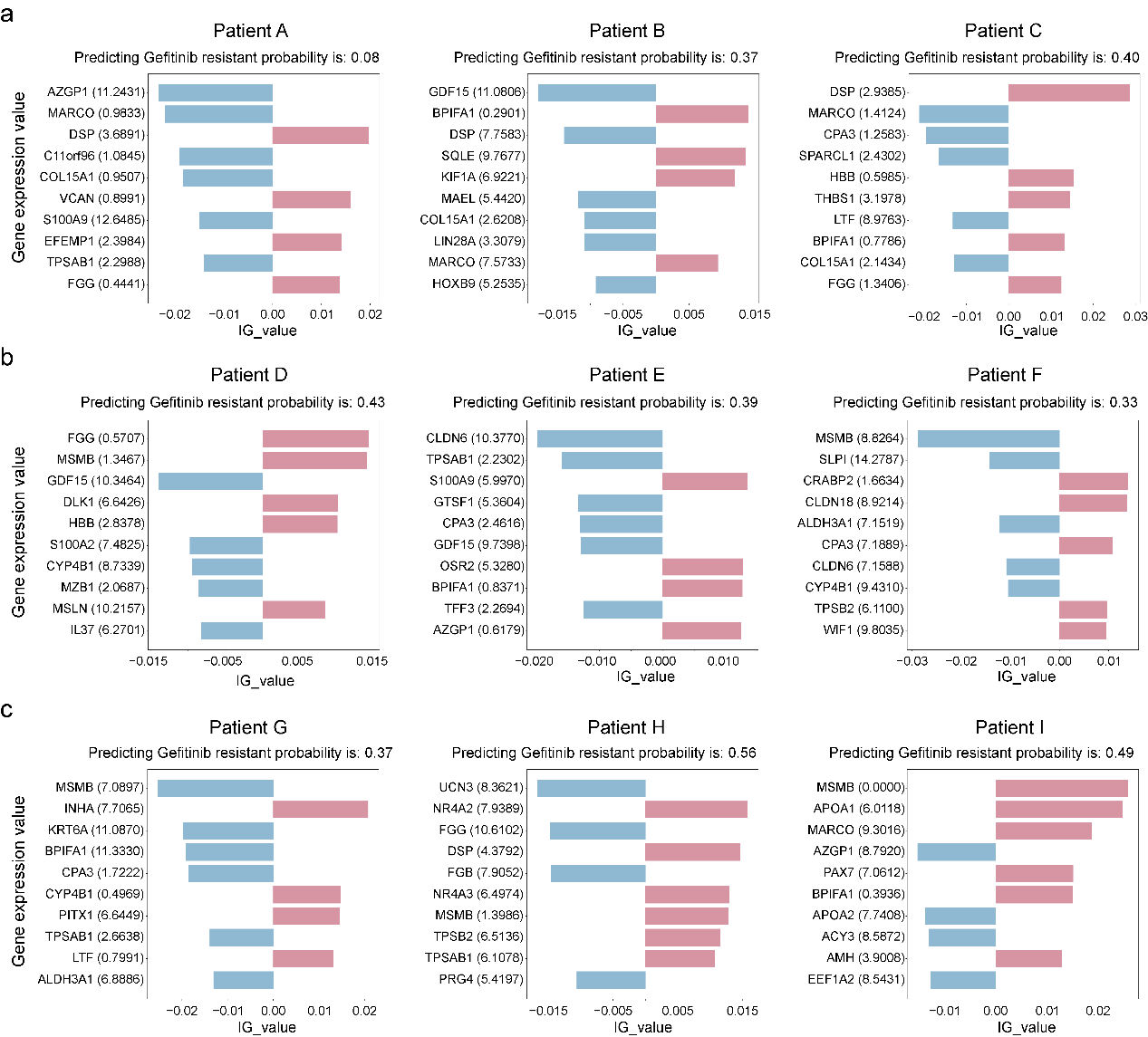
**

**Figure S9**. Results of individual patient gene contribution to drug resisentance. **(a).** Probability of Gefitinib resistance in OS-0-Max3 group. **(b).** Probability of Gefitinib resistance in OS-1-Max3 group **(c).** Probability of Gefitinib resistance in OS-1-Min3 group.
